# Incorporation of Shared Decision-Making in International Cardiovascular Guidelines, 2012-2022

**DOI:** 10.1001/jamanetworkopen.2023.32793

**Published:** 2023-09-07

**Authors:** Blair J. MacDonald, Ricky D. Turgeon

**Affiliations:** 1 Faculty of Pharmaceutical Sciences, University of British Columbia, Vancouver, British Columbia, Canada

## Abstract

**Importance:**

Shared decision-making (SDM) is a key component of the provision of ethical care, but prior reviews have indicated that clinical practice guidelines seldom promote or facilitate SDM. It is currently unknown whether these findings extend to contemporary cardiovascular guidelines.

**Objective:**

To identify and characterize integration of SDM in contemporary cardiovascular guideline recommendations using a systematic classification system.

**Design, Setting, and Participants:**

This cross-sectional study assessed the latest guidelines or subsequent updates that included pharmacotherapy recommendations and were published between January 2012 and December 2022 by the American College of Cardiology (ACC), Canadian Cardiovascular Society (CCS), and European Society of Cardiology (ESC). Data were analyzed from February 21 to July 21, 2023.

**Main Outcomes and Measures:**

All pharmacotherapy recommendations were identified within each guideline. Recommendations that incorporated SDM were rated according to a systematic rating framework to evaluate the quality of SDM incorporation based on directness (range, 1-3; assessing whether SDM was incorporated directly and impartially into the recommendation’s text, with 1 indicating direct and impartial incorporation of SDM into the recommendation’s text) and facilitation (range, A-D; assessing whether decision aids or quantified benefits and harms were provided, with A indicating that a decision aid quantifying benefits and harms was provided). The proportion of recommendations incorporating SDM was also analyzed according to guideline society and category (eg, general cardiology, heart failure).

**Results:**

Analyses included 65 guideline documents, and 33 documents (51%) incorporated SDM either in a general statement or within specific recommendations. Of 7499 recommendations, 2655 (35%) recommendations addressed pharmacotherapy, and of these, 170 (6%) incorporated SDM. By category, general cardiology guidelines contained the highest proportion of pharmacotherapy recommendations incorporating SDM (86 of 865 recommendations [10%]), whereas heart failure and myocardial disease contained the least (9 of 315 recommendations [3%]). The proportion of pharmacotherapy recommendations incorporating SDM was comparable across societies (ACC: 75 of 978 recommendations [8%]; CCS: 29 of 333 recommendations [9%]; ESC: 67 of 1344 recommendations [5%]), with no trend for change over time. Only 5 of 170 SDM recommendations (3%) were classified as grade 1A (impartial recommendations for SDM supported by a decision aid), whereas 114 of 170 recommendations (67%) were grade 3D (SDM mentioned only in supporting text and without any tools or information to facilitate SDM).

**Conclusions and Relevance:**

In this cross-sectional study across guidelines published by 3 major cardiovascular societies over the last decade, 51% of guidelines mentioned the importance of SDM, yet only 6% of recommendations incorporated SDM in any form, and fewer adequately facilitated SDM.

## Key Points

**Question:**

Is shared decision-making (SDM) integrated into cardiovascular guideline recommendations on pharmacotherapy?

**Findings:**

This cross-sectional study of 65 guidelines (2655 pharmacotherapy recommendations) published from 2012 to 2022 found that 6% of recommendations incorporated SDM in some form. The proportion of pharmacotherapy recommendations incorporating SDM was comparable across societies, with no trend for change over time, and only 3% of SDM recommendations were impartial and supported by a decision aid.

**Meaning:**

These findings suggest that SDM was infrequently promoted and facilitated across cardiovascular guidelines.

## Teaser

This cross-sectional study identifies and describes the integration of shared decision-making recommendations in contemporary cardiovascular clinical practice guidelines from 3 international societies.

## Introduction

Shared decision-making (SDM) is an essential aspect of the provision of ethical care, as it facilitates patient self-determination.^1,2,3^ Despite this, the current standard of care does not adequately inform nor involve patients in decision-making.^4,5,6,7,8,9^ For instance, a 2022 qualitative study of anticoagulant prescribing for atrial fibrillation^10^ found that clinicians provided unbalanced information regarding pros and cons, used persuasive language, were often unable to provide concrete cost information, and used direct-to-consumer advertising as an educational tool.

Clinical practice guidelines are well-positioned to promote and facilitate SDM, given their ubiquitous use to inform clinicians and policy makers. Guideline writers could help facilitate such promotion by including recommendations that advocate for SDM when multiple viable health care options are available.^11^ Furthermore, guideline writers could facilitate SDM by providing balanced, quantitative information about each treatment option, such as benefits, harms, and costs, or by encouraging use of evidence-based decision aids in their recommendations.^3,12,13,14,15^ Early studies aiming to quantify the extent of SDM incorporation in guidelines have identified substantial gaps, with less than 1% of the text dedicated to addressing the role of SDM in 5 major Canadian guidelines,^16^ and few prevention guidelines including any statements regarding patients’ values and preferences or providing quantitative estimates of benefits and harms.^17,18^

These studies suggest gaps and variability in the promotion and facilitation of SDM among guidelines; however, it is unknown whether these findings extend to a broad set of contemporary cardiovascular practice guidelines. The objectives of this study were to identify and characterize the integration of SDM, using a systematic classification system, among all pharmacotherapy recommendations within contemporary cardiovascular guidelines.

## Methods

This cross-sectional study is reported in accordance with the Strengthening the Reporting of Observational Studies in Epidemiology (STROBE) reporting guideline. Given that we used only published data without any patient-specific information, an ethics review board was deemed unnecessary for the study.

### Search Strategy and Eligibility Criteria

One author (B.J.M.) searched the guideline libraries of 3 cardiovascular societies for all guidelines published between January 1, 2012 and January 25, 2023: American College of Cardiology (ACC), Canadian Cardiovascular Society (CCS), and European Society of Cardiology (ESC). Documents not formally classified as guidelines (eg, clinical practice updates, position statements, expert consensus documents, appropriate-use criteria, and performance measures) were excluded, as these documents generally do not undergo a formal voting process or include clinical recommendations.

We reviewed the latest version of all comprehensive guidelines and any subsequent focused updates, and we included 65 documents^19,20,21,22,23,24,25,26,27,28,29,30,31,32,33,34,35,36,37,38,39,40,41,42,43,44,45,46,47,48,49,50,51,52,53,54,55,56,57,58,59,60,61,62,63,64,65,66,67,68,69,70,71,72,73,74,75,76,77,78,79,80,81,82,83^ with at least 1 pharmacotherapy recommendation. We focused on recommendations pertaining to pharmacotherapy, since such recommendations are predominantly amenable to SDM and were within the scope of content expertise of the authors.

### General Statements Promoting SDM

We performed a post hoc evaluation of overarching statements in support of SDM to further characterize the incorporation of SDM statements within guidelines as a whole. For a guideline to meet this criterion, it needed to contain at least 1 general statement (ie, statements broadly applicable across interventions and patient populations) expressing the significance of SDM or encouraging its implementation.

### Identifying Recommendations Integrating SDM

We reviewed all pharmacotherapy recommendations to identify those that integrated SDM, either directly or via supporting text, by searching each guideline document for the following keywords: *decision aid*, *discuss*, *informed decision*, *joining in*, *partake* or *partaking*, *partner*, *patient-centered*, *patient choice*, *patient contribution*, *patient education*, *patient goal*, *patient involvement*, *patient priorities*, *preference*, *values*, *shared decision*, and *sharing*. We also searched specifically for *www.* to identify links to online decision aids. Recommendations were classified as incorporating SDM if they acknowledged any role for patient values or preferences in the decision. One author (B.J.M.) screened all pharmacotherapy recommendations for SDM incorporation. The other author (R.D.T.) then independently audited a random sample of 300 (approximately 10%) pharmacotherapy recommendations, masked to the first author’s inclusions, along with all pharmacotherapy recommendations classified as incorporating SDM by the first reviewer. Disagreements were resolved via discussion until consensus was reached.

### Characterizing SDM Integration

To appraise the quality of SDM integration, we developed a systematic rating framework, which rates each recommendation in 2 dimensions: directness, which represents the extent to which SDM is explicitly recommended with impartiality to viable options; and facilitation, which represents the extent to which the recommendation enables the implementation of SDM. Directness was rated on a scale of 1 to 3 levels, with lower level indicating more direct recommendation. Facilitation was graded on a scale of A to D, with A indicating the best and D, the worst. The final rating framework is provided in the Table, and the first iteration of this system is available in eTable 1 in Supplement 1. Both authors developed the framework iteratively while initially reviewing recommendations and then rated each pharmacotherapy recommendation using the final rating framework. Disagreements were resolved via discussion until consensus was reached.

**Table.** Directness Level and Facilitation Grade of Shared Decision-Making Integration Into Guideline Recommendations Rating Framework

Rating	Exemplar recommendation		
	Study (recommending society)	Subject	Quote
**Directness level**			
1. Explicitly and impartially recommends SDM without making a recommendation for or against a specific course of action	Grundy et al,^32^ 2019 (ACC)	Blood cholesterol	“Before therapy [for dyslipidemia] is prescribed, a patient-clinician discussion should take place to promote shared decision-making and should include the potential for [atherosclerotic cardiovascular disease] risk-reduction benefit, adverse effects, drug-drug interactions, and patient preferences.”
2. SDM is incorporated into the recommendation’s text, but preference for a specific course of action is stated or implied	Otto et al,^35^ 2021 (ACC)	Valvular heart disease	“For pregnant women with mechanical prostheses who require a dose of warfarin ≤5 mg/d to maintain a therapeutic INR, continuation of warfarin for all 3 trimesters is reasonable after full discussion with the patient about risks and benefits.”
3. SDM is incorporated only in the supporting text	Hindricks et al,^72^ 2021 (ESC)	Atrial fibrillation	“Rhythm control therapy is recommended for symptom and [quality of life] improvement in symptomatic patients with [atrial fibrillation].”; supporting text: “General recommendations regarding active informed patient involvement in shared decision-making also apply for rhythm control strategies.”
**Facilitation grade**			
A. Provides or links to a patient decision aid	Grundy et al,^32^ 2019 (ACC)	Blood cholesterol	“In adults older than 75 years with diabetes mellitus, it may be reasonable to initiate statin therapy after a clinician–patient discussion of potential benefits and risks.”; supporting text included links to tools.
B. Provides balanced quantitative information to facilitate SDM (quantified presentation of ≥1 benefit and ≥1 harm)	Andrade et al,^55^ 2020 (CCS)	Atrial fibrillation	“We recommend that OAC be prescribed for most patients with [atrial fibrillation] and age 65 years or older or CHADS_2_ score ≥1.”; supporting text: “There is considerable scope for [shared decision-making]…In patients aged 65 years or older and without other risk factors for stroke, use of [vitamin K antagonists] decreased the annual risk of stroke from 2.1% to 0.7% while it increased the risk of major bleeding by approximately 0.5% per year to 1.0%...”
C. Provides partial quantitative information to facilitate SDM (only quantifies benefits or harms)	Otto et al,^35^ 2021 (ACC)	Valvular heart disease	“For pregnant women with mechanical prostheses who require a dose of warfarin >5 mg/d to maintain a therapeutic INR, dose-adjusted [low molecular-weight heparin] at least 2 times per day during the first trimester, followed by warfarin for the second and third trimesters, may be considered.”; supporting text: “Some women, after discussion with their physicians, may choose to substitute [low molecular weight heparin] for low dose warfarin during the first trimester to eliminate the risk of warfarin embryopathy. This choice improves fetal outcomes but at the cost of increased maternal thrombotic complications.”
D. Does not provide any quantitative information on benefits or harms	Collet et al,^73^ 2021 (ESC)	Acute coronary syndrome without persistent ST-segment elevation	“For patients with 1 non-sex stroke risk factor, [oral anticoagulation] should be considered and treatment may be individualized based on net clinical benefit and consideration of patient values and preferences.”

Abbreviations: ACC, American College of Cardiology; CCS, Canadian Cardiovascular Society; CHADS_2_, congestive heart failure, hypertension, age ≥75 years, diabetes, prior stroke or transient ischemic attack or thromboembolism (doubled); ESC, European Society of Cardiology; INR, international normalized ratio; OAC, oral anticoagulant.

### Data Extraction

For each included guideline, 1 author (B.J.M.) extracted the following data: lead author last name, year published, society, whether it was an update or standalone guideline, the condition of focus, page count, word count (introduction to conclusion), number of authors, number of authors with no reported conflict of interest, total number of recommendations, whether the guideline contained at least 1 general statement promoting SDM, and all pharmacotherapy recommendations and relevant supporting text. Guidelines were further categorized into the following groups, adapted from a prior study^84^: general cardiology; congenital, valvular, and aortic diseases; coronary artery disease; electrophysiology; and heart failure and myocardial disease.

### Statistical Analysis

We report the frequency and percentage of pharmacotherapy recommendations that incorporated SDM, along with the number of guidelines that included at least 1 pharmacotherapy recommendation incorporating SDM. We explored time-trends by comparing the proportion of pharmacotherapy recommendations that incorporated SDM by year (2012-2022), along with the total number of pharmacotherapy recommendations in the same year. Additionally, we assessed patterns based on condition category and guideline society (ACC, CCS, and ESC). We used Microsoft Excel (Version 2306) to perform all analyses. Data were analyzed from February 21 to July 21, 2023.

## Results

Between January 2012 and December 2022, the ACC, CCS, and ESC published a total of 74 guidelines, and we included 65 guidelines that incorporated at least 1 pharmacotherapy recommendation. There were a total of 7499 recommendations with 2655 pertaining to pharmacotherapy (eTable 2 in Supplement 1). The reviewed guidelines totaled 4758 pages (2 182 969 words) with 1487 authors, of whom 409 declared no conflict of interest (eTable 3 in Supplement 1).

### General Statements Supporting SDM

Of the 65 included guidelines,^19,20,21,22,23,24,25,26,27,28,29,30,31,32,33,34,35,36,37,38,39,40,41,42,43,44,45,46,47,48,49,50,51,52,53,54,55,56,57,58,59,60,61,62,63,64,65,66,67,68,69,70,71,72,73,74,75,76,77,78,79,80,81,82,83^ 33 guidelines^19,20,21,22,23,24,26,27,28,29,30,31,32,33,34,35,36,37,38,39,43,44,48,55,61,70,72,75,77,78,80,82,83^ (51%) included at least 1 statement expressing the importance of SDM or promoting its implementation. These 33 guidelines encompassed 1512 of 2655 pharmacotherapy recommendations (57%). The proportion of guidelines containing these general statements differed by guideline society (ACC: 20 of 21 guidelines [95%]; CCS: 9 of 28 guidelines [25%]; ESC: 4 of 16 guidelines [32%]).

### Recommendations That Integrated SDM

**Figure 1.**
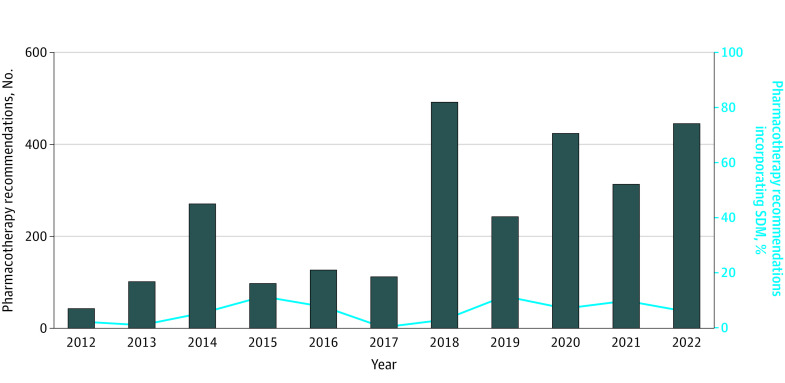
Proportion of Pharmacotherapy Recommendations Integrating Shared Decision-Making From American College of Cardiology, Canadian Cardiovascular Society, and European Society of Cardiology Guidelines by Year

**Figure 2.**
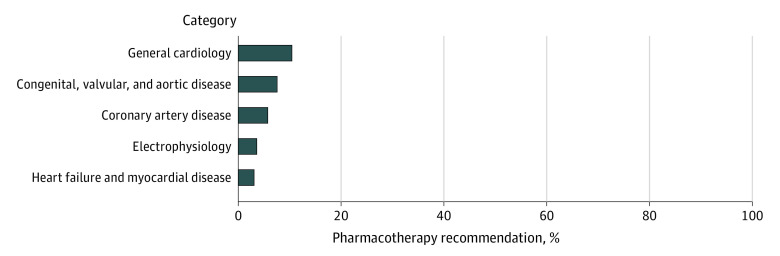
Proportion of 2012-2022 American College of Cardiology, Canadian Cardiovascular Society, and European Society of Cardiology Guideline Pharmacotherapy Recommendations Incorporating Shared Decision-Making by Category

**Figure 3.**
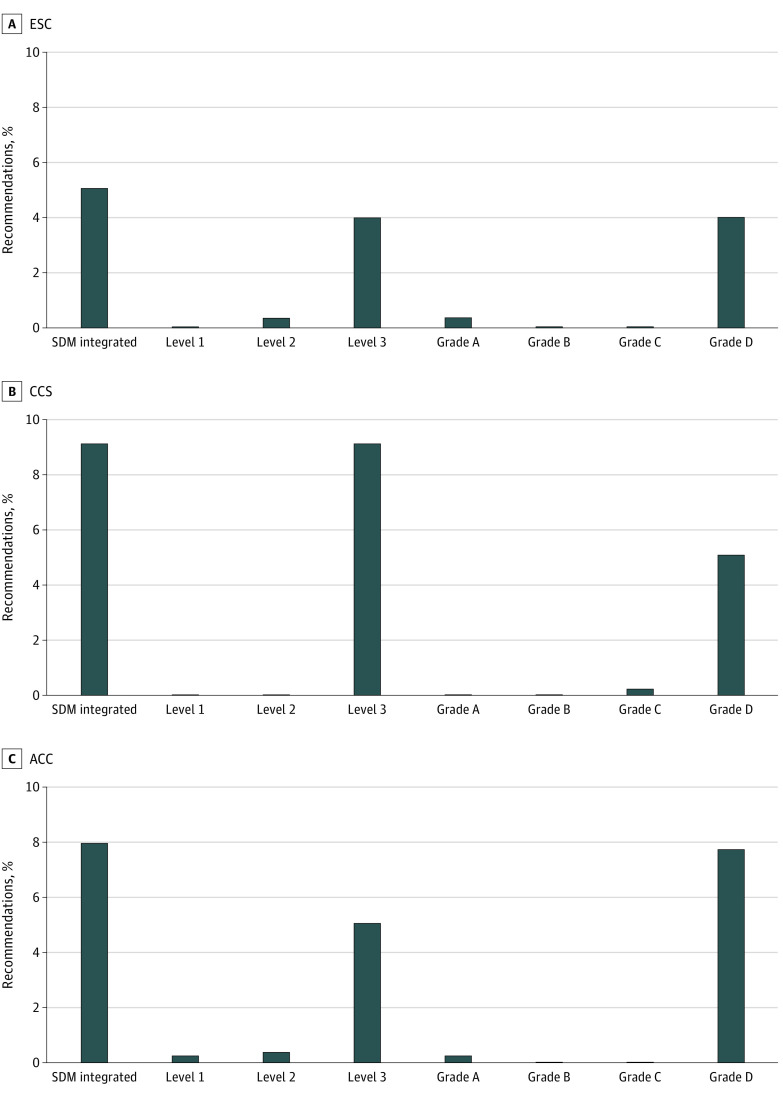
Proportion of 2012-2022 Guideline Pharmacotherapy Recommendations by Directness Level and Facilitation Grade by Society Grade A indicates that a patient decision aid was provided; grade B, balanced quantitative information was provided; grade C, partial quantitative information was provided; and grade D, no quantitative information provided. Level 1 indicates that guidelines explicitly and impartially recommended shared decision-making (SDM); level 2, SDM is incorporated into the recommendation’s text with stated or implied specific course of action; and level 3, SDM is incorporated only in the supporting text. ACC indicates American College of Cardiology; CCS, Canadian Cardiovascular Society; ESC, European Society of Cardiology.

A total of 170 recommendations (6%) from 29 guidelines^19,20,21,22,23,24,25,32,33,34,35,36,39,41,43,44,45,50,55,59,61,64,68,72,73,78,81,82^ incorporated SDM at some level. There was no apparent trend in this proportion over time (Figure 1). General cardiology guidelines contained the highest proportion of pharmacotherapy recommendations incorporating SDM (86 of 865 recommendations [10%]), whereas heart failure and myocardial disease (9 of 315 recommendations [3%]) contained the least (Figure 2). The 3 societies had a similar proportion of pharmacotherapy recommendations incorporating SDM (ACC: 75 of 978 recommendations [8%], CCS: 29 of 333 recommendations [9%], ESC: 67 of 1344 recommendations [5%]) (Figure 3). There was complete agreement between reviewers regarding whether the audited subset of pharmacotherapy recommendations incorporated SDM.

### Characterizing SDM Integration in Recommendations

**Figure 4.**
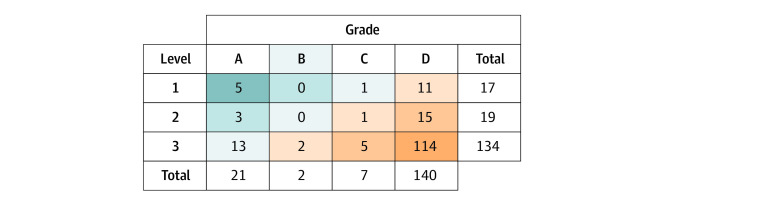
Characterization of Shared Decision-Making Integration in 170 Pharmacotherapy Recommendations Within 2012-2022 American College of Cardiology, Canadian Cardiovascular Society, and European Society of Cardiology Guidelines Dark blue indicates shared decision-making was directly integrated and facilitated; dark orange, shared decision-making integration was indirect without facilitation.

Figure 4 provides the directness level and facilitation grade of SDM incorporation. Of 170 pharmacotherapy recommendations integrating SDM, only 5 recommendations (3%) received the highest rating of 1A (impartial recommendations for SDM supported by a decision aid), while 114 recommendations (67%) received the lowest rating of 3D (SDM mentioned only in supporting text and without any tools or information to facilitate SDM). The ACC 2018 cholesterol guideline^32^ consistently and meaningfully incorporated SDM throughout their recommendations, with a large number of recommendations referring to a decision aid (grade A based on our framework). Across guideline societies, most recommendations were level 3 or grade D (Figure 3).

## Discussion

In this cross-sectional study of 65 cardiovascular guidelines published by 3 international cardiovascular societies, 51% broadly supported the importance of SDM, yet only 6% of pharmacotherapy recommendations incorporated SDM in some form. Among 170 recommendations that incorporated SDM, most merely noted the importance of patient preferences in the supporting text without addressing how to integrate these preferences into decision-making. These findings were consistent over the 10-year period and were similar across cardiovascular categories and between societies.

Despite these overall results, 2 identified guidelines exemplified commendable SDM incorporation. Specifically, 43% of the pharmacotherapy recommendations in the ACC 2018 cholesterol guideline incorporated SDM (approximately 7 times higher than the mean), with 32% of these recommendations receiving a grade A (indicating that a decision aid was provided).^32^ These recommendations are further supported by a general statement emphasizing the importance of SDM implementation. Additionally, the CCS 2022 cardiometabolic guideline reported a summary of findings table that could be used to facilitate SDM by providing estimates of benefit on patient-oriented efficacy outcomes.^44^

These exceptions aside, our overall findings echo and expand on the results of a 2007 review^16^ of major Canadian guidelines on diabetes, dyslipidemia, hypertension, and osteoporosis, in which only 0.1% of the total words in the guidelines related to SDM and patient preferences. Facilitation of SDM was likewise found to be poor, as necessary information was either missing or presented in an imbalanced manner. Similarly, a 2019 review^17^ found that only 39% of osteoporosis guidelines included any statements regarding patients’ beliefs, values, and preferences. A 2020 review^18^ of 13 coronary artery disease and diabetes guidelines found estimates of absolute benefit or harm in only 25% of recommendations, and only 3% included both a benefit and a harm. This indicates that these issues are widespread and have not noticeably improved over time.

The inclusion of broad statements supporting the principles and role of SDM in patient care is an important starting point, and the ACC’s guidelines are leaders in cardiovascular guidelines in this respect. Such broad statements serve as acknowledgments of the central role of patient values in decision-making but may not provide the support necessary for busy clinicians who are already faced with serious time constraints in implementing guideline-directed care.^85^ Several approaches could be undertaken to improve the incorporation of SDM into future guidelines. The Guidelines International Network has published a public toolkit for guideline development,^15^ in which 1 chapter is devoted to describing several possible SDM integration strategies. Some examples of these strategies include changing the wording of recommendations to promote discussion, presenting the absolute benefits and harms of interventions, and incorporating decision aids. The United Kingdom’s National Institute for Health and Care Excellence also recently published a guideline exclusively on how to integrate SDM into patient care across conditions and settings, as well as at an organizational level.^86^ Alternatively as a starting point, guideline panels could adopt a simplified approach. First, they could identify recommendations amenable to SDM, particularly weak and conditional recommendations that by their nature have at least 2 reasonable options. However, this should not preclude use of SDM in situations where strong recommendations are made. Second, recommendations that integrate SDM should be worded impartially with explicit incorporation of patient values and preferences, ideally referencing any existing evidence on patient preferences. Finally, guideline panels should provide information syntheses on the benefits, harms, and other considerations, such as cost, to help inform patient decisions, ideally in the form of a decision aid (pre-existing or created as part of guideline development). In absence of a bespoke decision aid, a Grading of Recommendations Assessment, Development and Evaluation summary of findings table outlining benefits and harms in absolute terms accompanying the recommendation can be used to facilitate SDM.^87^

Future research should also aim to better understand the reasons that guideline panels do not frequently incorporate SDM promotion or facilitation into individual recommendations. Such research should examine not only the barriers to incorporating SDM in this manner, but also the facilitators that enable certain guidelines to perform well on these metrics. Future research may also validate and incorporate this rating framework and use it to classify SDM incorporation in recommendations or monitor for changes over time. This longitudinal monitoring could assess the impact of the recently published SDM incorporation recommendations by the Guidelines International Network and National Institute for Health and Care Excellence,^86^ which will require further time for integration into guideline development.^15^ This framework may also serve as an effective complement to the AGREE II checklist,^88^ which is used to appraise guideline development processes and reporting, without direct examination of SDM incorporation.

### Limitations

This study has several limitations that warrant consideration. First, some of the included recommendations may not have been amenable to SDM (eg, recommendations pertaining to emergency situations or therapies with harm and no benefit); however, most of the included recommendations addressed chronic conditions with multiple viable treatment options. For example, among general cardiology guidelines, only 10% of pharmacotherapy recommendations incorporated SDM. Second, this method did not account for SDM integration found within incidental supporting text or supplemental documents unattached to the recommendations. Third, we did not perform a quality assessment of recommended decision aids and therefore did not differentiate between recommendations recommending high-quality from low-quality or unproven decision aids. Fourth, psychometric testing of the rating framework is ongoing, and interrater reliability among a broader set of raters is uncertain, though all SDM recommendations are provided in eTable 2 in Supplement 1 for readers to judge for themselves.

## Conclusions

This cross-sectional study found that across guidelines published by three major cardiovascular societies over the last decade, 51% of guidelines mentioned SDM, yet only 6% of recommendations incorporated SDM in any form, and even fewer adequately facilitated SDM. Future cardiovascular guidelines should further incorporate SDM into their recommendations to empower clinicians to engage patients in decision-making.

## Notes

Supplement 1. **eTable 1.** Early Version of Classification System **eTable 2.** List of Pharmacological Recommendations With Supporting Text Along With Final Rating **eTable 3.** List of Included Guidelines and Their Characteristics

Supplement 2.
Data Sharing Statement
