# Supplementary material for: Incorporation of Shared Decision-Making in International Cardiovascular Guidelines, 2012-2022
Source: JAMA Netw Open. 2023 Sep 7;6(9):e2332793. doi: 10.1001/jamanetworkopen.2023.32793 (PMC10485733; doi:10.1001/jamanetworkopen.2023.32793)
Supplement: Supplement 1. — eTable 1. Early Version of Classification System eTable 2. List of Pharmacological Recommendations With Supporting Text Along With Final Rating eTable 3. List of Included Guidelines and Their Characteristics [file jamanetwopen-e2332793-s001.pdf]

Supplemental Online Content

MacDonald BJ, Turgeon RD. Incorporation of shared decision-making in international cardiovascular guidelines, 2012-2022. *JAMA Netw Open*. 2023;6(9):e2332793. doi:10.1001/jamanetworkopen.2023.32793

eTable 1. Early Version of Classification System

eTable 2. List of Pharmacological Recommendations With Supporting Text Along With Final Rating

eTable 3. List of Included Guidelines and Their Characteristics

This supplemental material has been provided by the authors to give readers additional information about their work.

© 2023 MacDonald BJ et al. *JAMA Network Open.*

eTable 1. Early version of classification system

Level	Category	Definition
1a	Decision aid	Recommendation statement recommends the use of a specific decision aid to facilitate shared decision-making.
1b	Quantitative information	Recommendation statement or supporting text provides quantitative information about benefits and harms to allow for shared decision-making.
2a	Preferences and values	Description of a decision as being appropriate for shared decision-making, being preference-sensitive and describing qualitative studies or stated-preference studies on patient preferences/values relevant to the decision
2b	Shared decision	Recommendation statement that presents a decision as having no single best option and requiring patient involvement.
3 (lowest level)	General statement	General statement about the importance of integrating patient preferences and values into the decision without supporting literature on patient stated preferences, quantitative data on benefits and harms of options, or guidance on how to engage in shared decision-making.

© 2023 MacDonald BJ et al. *JAMA Network Open.*

eTable 2. List of pharmacological recommendations with supporting text along with final rating

Guideline (Year of Publication)	Recommendation	Supporting Text Incorporating Shared Decision Making	Directness	Facilitation
ESC 2021 Cardiovascular disease prevention	In apparently healthy people, after estimation of 10-year fatal and non-fatal CVD risk, lifetime risk and treatment benefit, risk modifiers, frailty, polypharmacy, and patient preferences should be considered		1	A
ESC 2021 Cardiovascular disease prevention	Treatment of ASCVD risk factors should be considered in apparently healthy people without DM, CKD, genetic/rarer lipid, or BP disorders who are at high CVD risk (SCORE2 2.5 to <7.5% for age under 50; SCORE2 5 to <10% for age 5069; SCORE2-OP 7.5 to <15% for age >70 years), taking CVD risk modifiers, lifetime risk and treatment benefit, and patient preferences into account.		1	A
ACC 2014 AF	In patients with AF, antithrombotic therapy should be individualized based on shared decision making after discussion of the absolute risks and RRs of stroke and bleeding and the patient's values and preferences	The selection of an antithrombotic agent should be based on shared decision making that takes into account risk factors, cost, tolerability, patient preference, potential for drug interactions, and other clinical characteristics, including time in the INR therapeutic range if the patient has been on warfarin<br><br>The new agents are currently considerably more expensive than warfarin. However, dietary limitations and the need for repeated INR testing are eliminated with the new agents. If patients are stable, their condition is easily controlled, and they are satisfied with warfarin therapy, it is not necessary to change to a new agent. However, it is important to discuss this option with patients who are candidates for the new agents.<br><br>[Link to no longer functional decision aid included]	1	A
ACC 2019 Cholesterol	Clinicians and patients should engage in a risk discussion that considers risk factors, adherence to healthy lifestyle, the potential for ASCVD risk-reduction benefits, and the potential for adverse effects and drug–drug interactions, as well as patient preferences, for an individualized treatment decision	The present guidelines continue to emphasize the importance of a clinician–patient risk discussion (S4.4.2-12—S4.4.2-14, S4.4.2-27, S4.4.2-28). In those with a 10-year ASCVD risk of ≥7.5%, it is recommended that the discussion occur before a statin prescription is written (S4.4.2-26). This frank discussion, as recommended in the 2013 ACC/AHA cholesterol guidelines (S4.4.2-26), should consider whether ASCVD risk factors have been addressed, evaluate whether an optimal lifestyle has been implemented, and review the potential for statin benefit versus the potential for adverse effects and drug-drug interactions. Then, on the basis of individual characteristics and including an	1	A

© 2023 MacDonald BJ et al. *JAMA Network Open.*

Guideline (Year of Publication)	Recommendation	Supporting Text Incorporating Shared Decision Making	Directness	Facilitation
		informed patient preference in shared decision-making, a risk decision about statin therapy can be made. Clinicians should indicate that as ASCVD risk increases, so does benefit of evidence-based LDL-C – lowering therapy. They may wish to review the drug and safety sections of the present guideline and stay informed on safety information that is essential for a balanced discussion. Importantly, for those at intermediate-risk, especially those > 55 years of age, risk-enhancing fac- tors or CAC can be used to clarify risk if the risk decision is uncertain (S4.4.2-16). Risk-enhancing factors, such as family history of premature ASCVD or an LDL-C of 160 to 189 mg/dL (4.1 – 4.8 mmol/L), identify individuals whose ASCVD risk may indicate risk of genetic hypercholesterolemia and hence who may benefit from a moderate-to high-intensity statin (S4.4.2-21) (Table 6)<br><br>Link to tools: https://statindecisionaid.mayoclinic.org/ https://tools.acc.org/ascvd-risk-estimator-plus/#!/calculate/estimate/		
ACC 2019 Cholesterol	Before therapy is prescribed, a patient-clinician discussion should take place to promote shared decision making and should include the potential for ASCVD risk-reduction benefit, adverse effects, drug-drug interactions, and patient preferences	Patients who participate in shared decision-making may have better health outcomes, better healthcare experiences, and lower costs (S6-7, S6-8). During the clinician–patient risk discussion with shared decision-making, the patient participates with the clinician in deciding lifestyle modifications, medication treatment, and goals of therapy. The clinician should explain the patient's risk of clinical ASCVD and how the treatment recommendations reduce ASCVD risk. The patient should verbalize values, attitudes, abilities, concerns, and personal goals for making lifestyle changes and taking medications, including concerns about cost (S6-22). The clinician may use a checklist to facilitate shared decision-making with the patient (Table S8 in the Web Supplement). See: http://jaccjacc.acc.org/Clinical_Document/ Cholesterol_GL_Web_Supplement.pdf<br><br>Link to tools: https://statindecisionaid.mayoclinic.org/ https://tools.acc.org/ascvd-risk-estimator-plus/#!/calculate/estimate/	1	A
ACC 2021 Valvular Heart Disease	Women with mechanical heart valves and their providers should use shared decision-making to choose an anticoagulation strategy for pregnancy. Women should be informed that VKA during pregnancy is associated with the lowest	Depending on a woman's values and priorities, she may choose an anticoagulation strategy that minimizes maternal risk, minimizes fetal risk, or attempts to achieve a balance between maternal and fetal risk. Physicians should not assume a woman's values or preferences, nor should physicians supplant	1	C

© 2023 MacDonald BJ et al. *JAMA Network Open.*

Guideline (Year of Publication)	Recommendation	Supporting Text Incorporating Shared Decision Making	Directness	Facilitation
	likelihood of maternal complications but the highest likelihood of miscarriage, fetal death, and congenital abnormalities, particularly if taken during the first trimester and if the warfarin dose exceeds 5 mg/d	their own preferences for those of the patient. Counseling and shared decision-making allows for a woman and her physician to choose the best anticoagulation to achieve the woman's goals		
ESC 2015 Pregnancy CV	In the absence of adequate human safety data, decision-making should be based on individual drug efficacy and safety profiles, and the available animal data, and the decision must be made together with the patient.		1	D
ESC 2022 Cardio-oncology	Discussion of the risk/benefit balance of cardiotoxic anticancer treatment in high- and very high-risk patients in a multidisciplinary approach prior to starting treatment is recommended.		1	D
ESC 2022 Cardio-oncology	A discussion with the patient about the relative benefits and harms, cancer prognosis, drug cost, and duration of treatment is recommended prior to prophylactic anticoagulation for the primary prevention of VTE.		1	D
ACC 2019 Atrial Fibrillation	In patients with AF, anticoagulant therapy should be individualized on the basis of shared decision making after discussion of the absolute risks and relative risks of stroke and bleeding, as well as the patient's values and preferences.		1	D
ACC 2019 Primary prevention	In adults at intermediate risk (≥7.5% to <20% 10-year ASCVD risk) or selected adults at borderline risk (5% to <7.5% 10-year ASCVD risk), if risk-based decisions for preventive interventions (e.g., statin therapy) remain uncertain, it is reasonable to measure a coronary artery calcium score to guide clinician– patient risk discussion	Coronary artery calcium measurement is not intended as a "screening" test for all but rather may be used as a decision aid in select adults to facilitate the clinician–patient risk discussion.	1	D
ACC 2020 Hypertrophic Cardiomyopathy	For patients with HCM or at risk for HCM, shared decision-making is recommended in developing a plan of care (including but not limited to decisions regarding genetic evaluation, activity, lifestyle, and therapy choices) that includes a full disclosure of the risks, benefits, and anticipated outcomes of all options, as well the opportunity for the patient to express their goals and concerns		1	D

© 2023 MacDonald BJ et al. *JAMA Network Open.*

Guideline (Year of Publication)	Recommendation	Supporting Text Incorporating Shared Decision Making	Directness	Facilitation
ACC 2020 Hypertrophic Cardiomyopathy	In adults with HCM and symptomatic ventricular arrhythmias or recurrent ICD shocks despite betablocker use, antiarrhythmic drug therapy listed is recommended, with the choice of agent guided by age, underlying comorbidities, severity of disease, patient preferences, and balance between efficacy and safety Amiodarone,		1	D
ACC 2020 Hypertrophic Cardiomyopathy	In adults with HCM and symptomatic ventricular arrhythmias or recurrent ICD shocks despite betablocker use, antiarrhythmic drug therapy listed is recommended, with the choice of agent guided by age, underlying comorbidities, severity of disease, patient preferences, and balance between efficacy and safety Dofetilide, Mexiletine, Sotalol		1	D
ACC 2020 Hypertrophic Cardiomyopathy	In children with HCM and recurrent ventricular arrhythmias despite beta-blocker use, antiarrhythmic drug therapy (amiodarone (3,4), mexiletine (6), sotalol (3,4)) is recommended, with the choice of agent guided by age, underlying comorbidities, severity of disease, patient preferences, and balance of efficacy and safety.		1	D
ACC 2020 Hypertrophic Cardiomyopathy	In patients with AF in whom rate control strategy is planned, either beta-blockers, verapamil, or diltiazem are recommended, with the choice of agents according to patient preferences and comorbid conditions		1	D
ACC 2020 Hypertrophic Cardiomyopathy	In patients with HCM and poorly tolerated AF, a rhythm control strategy with cardioversion or antiarrhythmic drugs can be beneficial with the choice of an agent according to AF symptom severity, patient preferences, and comorbid conditions		1	D
ACC 2019 Cholesterol	In adults older than 75 years with diabetes mellitus, it may be reasonable to initiate statin therapy after a clinician–patient discussion of potential benefits and risks	Although the risk of ASCVD is high in adults >75 years of age with diabetes mellitus (S4.3-5, S4.3-6, S4.3-8) who are not receiving statin therapy, particularly those with additional risk factors or risk modifiers, the benefit of initiating statin therapy in these individuals may be limited by their reduced life span or	2	A

© 2023 MacDonald BJ et al. *JAMA Network Open.*

Guideline (Year of Publication)	Recommendation	Supporting Text Incorporating Shared Decision Making	Directness	Facilitation
		increased susceptibility to adverse effects of treatment. Among this group will also be individuals with recent or newly diagnosed diabetes mellitus for whom the impact of diabetes mellitus on ASCVD risk is not well known. It may therefore be reasonable to have a clinician– patient discussion in which the potential benefits and risks of initiating statin therapy in this age group are reviewed.<br><br>Link to tools: https://statindecisionaid.mayoclinic.org/ https://tools.acc.org/ascvd-risk-estimator-plus/#!/calculate/estimate/		
ACC 2019 Cholesterol	In adults at intermediate-risk, statin therapy reduces risk of ASCVD, and in the context of a risk discussion, if a decision is made for statin therapy, a moderate-intensity statin should be recommended	Link to tools: https://statindecisionaid.mayoclinic.org/ https://tools.acc.org/ascvd-risk-estimator-plus/#!/calculate/estimate/	2	A
ACC 2019 Cholesterol	A clinician–patient risk discussion is recommended before initiation of statin therapy to review net clinical benefit, weighing the potential for ASCVD risk reduction against the potential for statin-associated side effects, statin–drug interactions, and safety, while emphasizing that side effects can be addressed successfully	A clinician–patient risk discussion focused on indications, benefits, risks of statin-associated side effects, and patient concerns and preferences should precede initiation of statin treatment<br><br>Link to tools: https://statindecisionaid.mayoclinic.org/ https://tools.acc.org/ascvd-risk-estimator-plus/#!/calculate/estimate/	2	A
ACC 2021 Valvular Heart Disease	For pregnant women with mechanical prostheses who require a dose of warfarin ≤5 mg/d to maintain a therapeutic INR, continuation of warfarin for all 3 trimesters is reasonable after full discussion with the patient about risks and benefits	The teratogenic effects of warfarin are dose dependent. The rate of warfarin embryopathy is reduced(<3%) but not eliminated if the daily dose of warfarin is 5 mg/d	2	C
ESC 2020 Atrial fibrillation	OAC should be considered for stroke prevention in AF patients with a CHA2DS2-VASc score of 1 in men or 2 in women. Treatment should be individualized based on net clinical benefit and consideration of patient values and preferences		2	D
ESC 2020 Atrial fibrillation	Long-term OAC therapy to prevent thromboembolic events should be considered in patients at risk for stroke with postoperative AF after non-cardiac surgery, considering the anticipated net clinical benefit of OAC therapy and informed patient preferences		2	D

© 2023 MacDonald BJ et al. *JAMA Network Open.*

Guideline (Year of Publication)	Recommendation	Supporting Text Incorporating Shared Decision Making	Directness	Facilitation
ESC 2020 Atrial fibrillation	Long-term OAC therapy to prevent thromboembolic events may be considered in patients at risk for stroke with postoperative AF after cardiac surgery, considering the anticipated net clinical benefit of OAC therapy and informed patient preferences		2	D
ESC 2021 ACS without STEMI	For patients with 1 non-sex stroke risk factor, OAC should be considered and treatment may be individualized based on net clinical benefit and consideration of patient values and preferences		2	D
ESC 2021 Cardiovascular disease prevention	A stepwise treatment-intensification approach aiming at intensive risk factor treatment is recommended for apparently healthy people at high or very high CVD risk, as well as patients with established ASCVD and/or DM, with consideration of CVD risk, treatment benefit of risk factors, risk modifiers, comorbidities, and patient preferences.	As not all drugs are tolerated or available/affordable, treatment should focus on achieving LDL-C levels as close as possible to the given goals. Treatment should be a shared decision-making process between physicians and the patient.	2	D
ESC 2021 Cardiovascular disease prevention	A stepwise treatment-intensification approach is recommended for apparently healthy people at high or very high CVD risk, as well as patients with established ASCVD and/or DM with consideration of CVD risk, treatment benefit, risk modifiers, comorbidities, and patient preferences.	As not all drugs are tolerated or available/affordable, treatment should focus on achieving LDL-C levels as close as possible to the given goals. Treatment should be a shared decision-making process between physicians and the patient.	2	D
ESC 2022 Cardio-oncology	In patients with cancer and pre-existing severe VHD, management according to the 2021 ESC/ EACTS Guidelines for the management of VHD is recommended, taking into consideration cancer prognosis and patient preferences	Cancer treatment should be temporarily interrupted, and an urgent multidisciplinary approach5 is indicated to plan an individualized guideline-based management, taking into account cancer status, prognosis, and the patient's preferences regarding invasive management.	2	D
ESC 2022 Cardiovascular assessment and manage of patients undergoing non-cardiac surgery	In patients with post-operative AF after NCS, long-term OAC therapy should be considered in all patients at risk of stroke, considering the anticipated net clinical benefit of OAC therapy, and informed patient preferences		2	D
ACC 2018 Hypertension	For older adults (‡65 years of age) with hypertension and a high burden of comorbidity and limited life expectancy, clinical judgment,		2	D

© 2023 MacDonald BJ et al. *JAMA Network Open.*

Guideline (Year of Publication)	Recommendation	Supporting Text Incorporating Shared Decision Making	Directness	Facilitation
	patient preference, and a team-based approach to assess risk/benefit is reasonable for decisions regarding intensity of BP lowering and choice of antihypertensive drugs			
ACC 2019 Cholesterol	In patients with clinical ASCVD who are judged to be very high risk and who are on maximally tolerated LDL-C lowering therapy with LDL-C 70 mg/dL or higher (≥1.8 mmol/L) or a non–HDL-C level of 100 mg/dL or higher (≥2.6 mmol/L) it is reasonable to add a PCSK9 inhibitor following a clinician–patient discussion about the net benefit, safety, and cost		2	D
ACC 2019 Cholesterol	In patients at borderline risk, in risk discussion, the presence of risk-enhancing factors may justify initiation of moderate-intensity statin therapy	This risk group benefits greatly from a clinician–patient risk discussion. To arrive at a shared risk decision, clinicians should assess the patient's priorities for health care, perceived ASCVD risk, and prior risk-reduction experiences and should use best practices to communicate numerical risk (S4.4.2-27). The presence of risk-enhancing factors provides useful information about short term ASCVD risk favoring initiation of statin therapy (Table 6)(S4.4.2-58). Although a CAC score can be useful in selected individuals, it will be positive less often in this lower-risk group than in those with higher levels of ASCVD risk and is not recommended routinely (S4.4.2-17) .	2	D
ACC 2019 Cholesterol	In adults 40 to 75 years of age with LDL-C 70 to 189 mg/dL (1.7 to 4.8 mmol/L) who have a 10-year ASCVD risk of 7.5% or higher, chronic inflammatory disorders and HIV are risk-enhancing factors and in risk discussion favor moderate-intensity statin therapy or high-intensity statin therapy		2	D
ACC 2019 Primary prevention	In adults at intermediate risk (≥7.5% to <20% 10-year ASCVD risk), statin therapy reduces risk of ASCVD, and in the context of a risk discussion, if a decision is made for statin therapy, a moderate-intensity statin should be recommended		2	D
ACC 2019 Primary prevention	In patients at borderline risk (5% to <7.5% 10-year ASCVD risk), in risk discussion, the presence of risk enhancing factors may justify initiation of moderate-intensity statin therapy	This risk group benefits greatly from a clinician–patient risk discussion. Clinicians should assess priorities for health care, perceived ASCVD risk, and prior risk reduction experiences and should use best practices for communicating risk to arrive at a shared risk decision.	2	D

© 2023 MacDonald BJ et al. *JAMA Network Open.*

Guideline (Year of Publication)	Recommendation	Supporting Text Incorporating Shared Decision Making	Directness	Facilitation
ACC 2022 Heart failure	For patients with HF being considered for, or treated with, life-extending therapies, the option for discontinuation should be anticipated and discussed through the continuum of care, including at the time of initiation, and reassessed with changing medical conditions and shifting goals of care	Patients have a right to decline or withdraw care at any time, consistent with the principle of respect for autonomy	2	D
ESC 2021 Cardiovascular disease prevention	An ultimate LDL-C goal of <1.4 mmol/L (55 mg/dL) and LDL-C reduction of >50% from baseline should be considered in apparently healthy persons <70 years at very high risk.	Figure 6 incorporates patient preferences into the algorithm Includes link to: www.u-prevent.com	3	A
ESC 2021 Cardiovascular disease prevention	An ultimate LDL-C goal of <1.8 mmol/L (70 mg/dL) and LDL-C reduction of >50% from baseline should be considered in apparently healthy persons <70 years at high risk	Figure 6 incorporates patient preferences into the algorithm Includes link to: www.u-prevent.com	3	A
ESC 2021 Cardiovascular disease prevention	In patients with established ASCVD, lipid-lowering treatment with an ultimate LDL-C goal of <1.4 mmol/L (55 mg/dL) and a >50% reduction in LDLC vs. baseline is recommended	Figure 7 incorporates patient preferences into the algorithm Includes link to: www.u-prevent.com	3	A
ESC 2021 Cardiovascular disease prevention	Initiation of statin treatment for primary prevention in older people aged >70 may be considered, if at high risk or above	Figure 6 incorporates patient preferences into the algorithm Includes link to: www.u-prevent.com	3	A
ESC 2021 Cardiovascular disease prevention	In patients with type 2 DM at very high risk (e.g. with established ASCVD and/or severe TOD), intensive lipid-lowering therapy, ultimately aiming at >50% LDL-C reduction and an LDL-C of <1.4 mmol/L (55 mg/dL) is recommended	Figure 8 incorporates patient preferences into the algorithm Includes link to: www.u-prevent.com	3	A
ESC 2021 Cardiovascular disease prevention	For grade 1 hypertension, treatment initiation based on absolute CVD risk, estimated lifetime benefit, and the presence of HMOD is recommended	Figure 6 incorporates patient preferences into the algorithm Includes link to: www.u-prevent.com	3	A
ESC 2021 Cardiovascular disease prevention	For patients with grade 2 hypertension or higher, drug treatment is recommended.	Figure 6 incorporates patient preferences into the algorithm Includes link to: www.u-prevent.com	3	A
ESC 2021 Cardiovascular disease prevention	It is recommended that the first objective of treatment is to lower BP to <140/90 mmHg in all patients, and that subsequent BP targets are tailored to age and specific comorbidities	Figure 6 incorporates patient preferences into the algorithm Includes link to: www.u-prevent.com	3	A
ESC 2021 Cardiovascular	In treated patients aged 18-69 years, it is recommended that SBP should ultimately be	Figure 6 incorporates patient preferences into the algorithm Includes link to: www.u-prevent.com	3	A

© 2023 MacDonald BJ et al. *JAMA Network Open.*

Guideline (Year of Publication)	Recommendation	Supporting Text Incorporating Shared Decision Making	Directness	Facilitation
disease prevention	lowered to a target range of 120 - 130 mmHg in most patients			
ESC 2021 Cardiovascular disease prevention	In treated patients aged >70 years, it is recommended that SBP should generally be targeted to <140 and down to 130 mmHg if tolerated	Figure 6 incorporates patient preferences into the algorithm<br>Includes link to: www.u-prevent.com	3	A
ACC 2014 AF	Selection of antithrombotic therapy should be based on the risk of thromboembolism irrespective of whether the AF pattern is paroxysmal, persistent, or permanent	The selection of an antithrombotic agent should be based on shared decision making that takes into account risk factors, cost, tolerability, patient preference, potential for drug interactions, and other clinical characteristics, including time in the INR therapeutic range if the patient has been on warfarin<br><br>The new agents are currently considerably more expensive than warfarin. However, dietary limitations and the need for repeated INR testing are eliminated with the new agents. If patients are stable, their condition is easily controlled, and they are satisfied with warfarin therapy, it is not necessary to change to a new agent. However, it is important to discuss this option with patients who are candidates for the new agents.<br><br>[Link to no longer functional decision aid included]	3	A
ACC 2019 Cholesterol	In intermediate-risk or selected borderline-risk adults, if the decision about statin use remains uncertain, it is reasonable to use a CAC score in the decision to withhold, postpone or initiate statin therapy	Assess CAC (Section 4.4.1.4.) if risk decision is uncertain and additional information is needed to clarify ASCVD risk. Use decision tools to explain risk (e.g., ASCVD Risk Estimator Plus,* Mayo Clinic Statin Choice Decision Aid†).	3	A
ACC 2019 Cholesterol	In adults 75 years of age or older with an LDL-C level of 70 to 189 mg/dL (1.7 to 4.8 mmol/L), initiating a moderate-intensity statin may be reasonable	Thus, clinician–patient discussion of risk versus benefit remains particularly important with inconsistent support and few data for adults >80 years of age.<br><br>Link to tools: https://statindecisionaid.mayoclinic.org/<br>https://tools.acc.org/ascvd-risk-estimator-plus/#!/calculate/estimate/	3	A
ACC 2021 Valvular Heart Disease	For patients with a thrombosed left-sided mechanical prosthetic heart valve who present with symptoms of valve obstruction, urgent initial treatment with either slow-infusion, low-dose fibrinolytic therapy or emergency surgery is recommended	The decision between surgery and systemic fibrinolysis for symptomatic left-sided mechanical valve thrombosis should be individualized (Table 23) after review by the heart valve team, while engaging the patient in a process of shared decision-making and accounting for local experience and expertise. The overall 30-day mortality rate with surgery is 10% to 15%, with a lower mortality rate of <5% in patients with NYHA class I or II symptoms (2,3,7). Recent studies using an echocardiogram-guided, slow-infusion, low-dose fibrinolytic protocol have shown	3	B

© 2023 MacDonald BJ et al. *JAMA Network Open.*

Guideline (Year of Publication)	Recommendation	Supporting Text Incorporating Shared Decision Making	Directness	Facilitation
		hemodynamic success rates >90%, with embolic event rates <2% and major bleeding rates <2% (13). Systemic fibrinolysis is therefore an acceptable alternative to reoperation in patients at high or prohibitive surgical risk and in patients who have a small thrombus burden, mild HF symptoms (NYHA class I or II), and low bleeding risk. Absence of surgical expertise should be considered in the clinical decision-making process as a factor that favors thrombolytics, whereas recurrent valve thrombosis favors a surgical approach		
CCS 2020 Atrial fibrillation	We recommend that OAC be prescribed for most patients with AF and age 65 years or older or CHADS2 score ≥1	The benefit of OAC must be weighed against the risk of hemorrhage. The relative importance of a stroke prevented, and a major bleed caused is a subjective judgement. There is considerable scope for physician-patient discussion (ie, shared decision-making) to ensure that patient values are concordant with the decision to prescribe OAC, particularly when the annual risk of stroke is < 2% per year. The CCS rationale for recommending OAC for most patients with age 65 years or older or CHADS2 score ≥1 is on the basis of the effects of OAC on the absolute risk reduction of stroke compared with the increase in major hemorrhage. In patients aged 65 years or older and without other risk factors for stroke, use of VKAs decreased the annual risk of stroke from 2.1% to 0.7% while it increased the risk of major bleeding by approximately 0.5% per year to 1.0%. Although the risk of major bleeding increases with increasing CHADS2 scores, the rate of rise is not as steep as that for stroke; therefore, the benefit to risk ratio for OAC increases as stroke risk factors accumulate. Furthermore, 70% of strokes result in death or major disability, whereas most patients survive major hemorrhage without long-term effects.196 Thus, these results favour use of OAC in patients with age 65 years or older or CHADS2 score 1. OACs with efficacy and safety evidence in AF include VKAs and DOACs.	3	B
ACC 2021 Valvular Heart Disease	For pregnant women with mechanical prostheses who require a dose of warfarin >5 mg/d to maintain a therapeutic INR, dose-adjusted LMWH at least 2 times per day during the first trimester, followed by warfarin for the second and third trimesters, may be considered	Some women, after discussion with their physicians, may choose to substitute LMWH for low dose warfarin during the first trimester to eliminate the risk of warfarin embryopathy. This choice improves fetal outcomes but at the cost of increased maternal thrombotic complications.	3	C
CCS 2020 Atrial fibrillation	We suggest that no antithrombotic therapy be prescribed for stroke prevention for most patients with NVAF who are aged younger than 65 years with no CHADS2 risk factors	The benefit of OAC must be weighed against the risk of hemorrhage. The relative importance of a stroke prevented, and a major bleed caused is a subjective judgement. There is considerable scope for physician-patient discussion (ie, shared	3	C

© 2023 MacDonald BJ et al. *JAMA Network Open.*

Guideline (Year of Publication)	Recommendation	Supporting Text Incorporating Shared Decision Making	Directness	Facilitation
		decision-making) to ensure that patient values are concordant with the decision to prescribe OAC, particularly when the annual risk of stroke is < 2% per year. The CCS rationale for recommending OAC for most patients with age 65 years or older or CHADS2 score ≥1 is on the basis of the effects of OAC on the absolute risk reduction of stroke compared with the increase in major hemorrhage. In patients aged 65 years or older and without other risk factors for stroke, use of VKAs decreased the annual risk of stroke from 2.1% to 0.7% while it increased the risk of major bleeding by approximately 0.5% per year to 1.0%. Although the risk of major bleeding increases with increasing CHADS2 scores, the rate of rise is not as steep as that for stroke; therefore, the benefit to risk ratio for OAC increases as stroke risk factors accumulate. Furthermore, 70% of strokes result in death or major disability, whereas most patients survive major hemorrhage without long-term effects.196 Thus, these results favour use of OAC in patients with age 65 years or older or CHADS2 score 1. OACs with efficacy and safety evidence in AF include VKAs and DOACs.		
CCS 2022 GLP1/SGLT2i	In adults with T2D and either established ASCVD or multiple risk factors for ASCVD, we recommend use of: a. GLP-1RA or SGLT2i to reduce the risk of all-cause or CV mortality or MACE	Finally, an individualized approach to therapy should also weigh the individual's preferences, costs and coverage, side effect profile, consideration of kidney function and glucose-lowering efficacy, desire for weight loss, and comorbidities such as frailty See Table 1	3	C
CCS 2022 GLP1/SGLT2i	In adults with T2D and either established ASCVD or multiple risk factors for ASCVD, we recommend use of: SGLT2i to reduce the risk of hospitalization for HF or the composite of significant decline in eGFR, progression to end stage kidney disease or kidney death	Finally, an individualized approach to therapy should also weigh the individual's preferences, costs and coverage, side effect profile, consideration of kidney function and glucose-lowering efficacy, desire for weight loss, and comorbidities such as frailty See Table 1	3	C
CCS 2022 GLP1/SGLT2i	In adults with T2D and either established ASCVD or multiple risk factors for ASCVD, we recommend use of: GLP-1RA to reduce the risk of nonfatal stroke	Finally, an individualized approach to therapy should also weigh the individual's preferences, costs and coverage, side effect profile, consideration of kidney function and glucose-lowering efficacy, desire for weight loss, and comorbidities such as frailty See Table 1	3	C
ESC 2015 Infective endocarditis	Antibiotic prophylaxis should be considered for patients at highest risk for IE: (1) Patients with any prosthetic valve, including a transcatheter valve, or those in whom any prosthetic material was used for cardiac valve repair.	Although this section of the guidelines on IE prophylaxis is based on weak evidence, they have been strengthened recently by epidemiological surveys, most of which did not show an increased incidence of IE due to oral streptococci. Their application by patients should follow a shared decision-making process.	3	D

© 2023 MacDonald BJ et al. *JAMA Network Open.*

Guideline (Year of Publication)	Recommendation	Supporting Text Incorporating Shared Decision Making	Directness	Facilitation
	(2) Patients with a previous episode of IE.<br>(3) Patients with CHD:<br>(a) Any type of cyanotic CHD.<br>(b) Any type of CHD repaired with a prosthetic material, whether placed surgically or by percutaneous techniques, up to 6 months after the procedure or lifelong if residual shunt or valvular regurgitation remains.			
ESC 2015 Infective endocarditis	Antibiotic prophylaxis is not recommended in other forms of valvular or CHD.	Although this section of the guidelines on IE prophylaxis is based on weak evidence, they have been strengthened recently by epidemiological surveys, most of which did not show an increased incidence of IE due to oral streptococci. Their application by patients should follow a shared decision-making process.	3	D
ESC 2015 Infective endocarditis	Antibiotic prophylaxis should only be considered for dental procedures requiring manipulation of the gingival or periapical region of the teeth or perforation of the oral mucosa	Although this section of the guidelines on IE prophylaxis is based on weak evidence, they have been strengthened recently by epidemiological surveys, most of which did not show an increased incidence of IE due to oral streptococci. Their application by patients should follow a shared decision-making process.	3	D
ESC 2015 Infective endocarditis	Antibiotic prophylaxis is not recommended for local anaesthetic injections in non-infected tissues, treatment of superficial caries, removal of sutures, dental X-rays, placement or adjustment of removable prosthodontic or orthodontic appliances or braces or following the shedding of deciduous teeth or trauma to the lips and oral mucosa	Although this section of the guidelines on IE prophylaxis is based on weak evidence, they have been strengthened recently by epidemiological surveys, most of which did not show an increased incidence of IE due to oral streptococci. Their application by patients should follow a shared decision-making process.	3	D
ESC 2015 Infective endocarditis	Antibiotic prophylaxis is not recommended for respiratory tract procedures, including bronchoscopy or laryngoscopy, or transnasal or endotracheal intubation	Although this section of the guidelines on IE prophylaxis is based on weak evidence, they have been strengthened recently by epidemiological surveys, most of which did not show an increased incidence of IE due to oral streptococci. Their application by patients should follow a shared decision-making process.	3	D
ESC 2015 Infective endocarditis	Antibiotic prophylaxis is not recommended for gastroscopy, colonoscopy, cystoscopy, vaginal or caesarean delivery or TOE	Although this section of the guidelines on IE prophylaxis is based on weak evidence, they have been strengthened recently by epidemiological surveys, most of which did not show an increased incidence of IE due to oral streptococci. Their application by patients should follow a shared decision-making process.	3	D

© 2023 MacDonald BJ et al. *JAMA Network Open.*

Guideline (Year of Publication)	Recommendation	Supporting Text Incorporating Shared Decision Making	Directness	Facilitation
ESC 2015 Infective endocarditis	Antibiotic prophylaxis is not recommended for any procedure	Although this section of the guidelines on IE prophylaxis is based on weak evidence, they have been strengthened recently by epidemiological surveys, most of which did not show an increased incidence of IE due to oral streptococci. Their application by patients should follow a shared decision-making process.	3	D
ESC 2015 Infective endocarditis	Perioperative prophylaxis is recommended before placement of a pacemaker or implantable cardioverter defibrillator	Although this section of the guidelines on IE prophylaxis is based on weak evidence, they have been strengthened recently by epidemiological surveys, most of which did not show an increased incidence of IE due to oral streptococci. Their application by patients should follow a shared decision-making process.	3	D
ESC 2015 Infective endocarditis	Potential sources of sepsis should be eliminated ≥2 weeks before implantation of a prosthetic valve or other intracardiac or intravascular foreign material, except in urgent procedures	Although this section of the guidelines on IE prophylaxis is based on weak evidence, they have been strengthened recently by epidemiological surveys, most of which did not show an increased incidence of IE due to oral streptococci. Their application by patients should follow a shared decision-making process.	3	D
ESC 2015 Infective endocarditis	Perioperative antibiotic prophylaxis should be considered in patients undergoing surgical or transcatheter implantation of a prosthetic valve, intravascular prosthetic or other foreign material	Although this section of the guidelines on IE prophylaxis is based on weak evidence, they have been strengthened recently by epidemiological surveys, most of which did not show an increased incidence of IE due to oral streptococci. Their application by patients should follow a shared decision-making process.	3	D
ESC 2015 Infective endocarditis	Systematic local treatment without screening of S. aureus is not recommended	Although this section of the guidelines on IE prophylaxis is based on weak evidence, they have been strengthened recently by epidemiological surveys, most of which did not show an increased incidence of IE due to oral streptococci. Their application by patients should follow a shared decision-making process.	3	D
ESC 2018 STEMI	The use of the polypill and combination therapy to increase adherence to drug therapy may be considered	Although low adherence has been qualified as an ubiquitous problem, healthcare professionals and patients should be aware of this challenge and optimize communication by providing clear information, simplify treatment regimens, aim at shared decision-making, and implement repetitive monitoring and feedback.	3	D
ESC 2020 Atrial fibrillation	Rhythm control therapy is recommended for symptom and QoL improvement in symptomatic patients with AF	General recommendations regarding active informed patient involvement in shared decision making also apply for rhythm control strategies	3	D

© 2023 MacDonald BJ et al. *JAMA Network Open.*

Guideline (Year of Publication)	Recommendation	Supporting Text Incorporating Shared Decision Making	Directness	Facilitation
ESC 2020 Chronic coronary syndromes	Medical treatment of symptomatic patients requires one or more drug(s) for angina/ischaemia relief in association with drug(s) for event prevention.	However, there is no universal definition of an optimal treatment in patients with CCS, and drug therapies must be adapted to each patient's characteristics and preferences	3	D
ESC 2020 Chronic coronary syndromes	Short-acting nitrates are recommended for immediate relief of effort angina	However, there is no universal definition of an optimal treatment in patients with CCS, and drug therapies must be adapted to each patient's characteristics and preferences	3	D
ESC 2020 Chronic coronary syndromes	First-line treatment is indicated with beta-blockers and/or CCBs to control heart rate and symptoms	However, there is no universal definition of an optimal treatment in patients with CCS, and drug therapies must be adapted to each patient's characteristics and preferences	3	D
ESC 2020 Chronic coronary syndromes	If angina symptoms are not successfully controlled on a beta-blocker or a CCB, the combination of a beta-blocker with a DHP-CCB should be considered.	However, there is no universal definition of an optimal treatment in patients with CCS, and drug therapies must be adapted to each patient's characteristics and preferences	3	D
ESC 2020 Chronic coronary syndromes	Initial first-line treatment with the combination of a beta-blocker and a DHP-CCB should be considered	However, there is no universal definition of an optimal treatment in patients with CCS, and drug therapies must be adapted to each patient's characteristics and preferences	3	D
ESC 2020 Chronic coronary syndromes	Long-acting nitrates should be considered as a second-line treatment option when initial therapy with a beta-blocker and/or a non-DHP-CCB is contraindicated, poorly tolerated, or inadequate to control angina symptoms	However, there is no universal definition of an optimal treatment in patients with CCS, and drug therapies must be adapted to each patient's characteristics and preferences	3	D
ESC 2020 Chronic coronary syndromes	When long-acting nitrates are prescribed, a nitrate-free or low-nitrate interval should be considered to reduce tolerance	However, there is no universal definition of an optimal treatment in patients with CCS, and drug therapies must be adapted to each patient's characteristics and preferences	3	D
ESC 2020 Chronic coronary syndromes	Nicorandil, ranolazine, ivabradine, or trimetazidine should be considered as a second-line treatment to reduce angina frequency and improve exercise tolerance in subjects who cannot tolerate, have contraindications to, or whose symptoms are not adequately controlled by beta-blockers, CCBs, and long-acting nitrates.	However, there is no universal definition of an optimal treatment in patients with CCS, and drug therapies must be adapted to each patient's characteristics and preferences	3	D
ESC 2020 Chronic coronary syndromes	In subjects with baseline low heart rate and low BP, ranolazine or trimetazidine may be considered as a first-line drug to reduce angina frequency and improve exercise tolerance	However, there is no universal definition of an optimal treatment in patients with CCS, and drug therapies must be adapted to each patient's characteristics and preferences	3	D

© 2023 MacDonald BJ et al. *JAMA Network Open.*

Guideline (Year of Publication)	Recommendation	Supporting Text Incorporating Shared Decision Making	Directness	Facilitation
ESC 2020 Chronic coronary syndromes	In selected patients, the combination of a beta-blocker or a CCB with second-line drugs (ranolazine, nicorandil, ivabradine, and trimetazidine) may be considered for first-line treatment according to heart rate, BP, and tolerance	However, there is no universal definition of an optimal treatment in patients with CCS, and drug therapies must be adapted to each patient's characteristics and preferences	3	D
ESC 2020 Chronic coronary syndromes	Nitrates are not recommended in patients with hypertrophic obstructive cardiomyopathy or co-administration of phosphodiesterase inhibitors	However, there is no universal definition of an optimal treatment in patients with CCS, and drug therapies must be adapted to each patient's characteristics and preferences	3	D
ESC 2021 ACS without STEMI	It is recommended to apply the same interventional strategies in older patients as for younger patients	Decisions as to how to manage older patients should be based on ischaemic and bleeding risks, estimated life expectancy, comorbidities, the need for non-cardiac surgery, quality of life, frailty, cognitive and functional impairment, patient values and preferences, and the estimated risks and benefits of revascularization	3	D
ESC 2021 Cardiovascular disease prevention	In all treated patients, DBP is recommended to be lowered to <80 mmHg	Figure 6 incorporates patient preferences into the algorithm Includes link to: www.u-prevent.com	3	D
ESC 2021 Cardiovascular disease prevention	In patients with type 2 DM but without ASCVD, HF, or CKD, use of an SGLT2 inhibitor or GLP-1RA should be considered based on estimated future risks (e.g. with the ADVANCE risk score or DIAL model) for adverse CVD or cardiorenal outcomes from risk factor profiles	Figure 8 incorporates patient preferences into the algorithm Includes link to: www.u-prevent.com	3	D
ESC 2021 Cardiovascular disease prevention	Low-dose colchicine (0.5 mg o.d.) may be considered in secondary prevention of CVD, particularly if other risk factors are insufficiently controlled or if recurrent CVD events occur under optimal therapy	Figure 7 incorporates patient preferences into the algorithm Includes link to: www.u-prevent.com	3	D
ESC 2021 Cardiovascular disease prevention	Adding a second antithrombotic drug (a P2Y12 inhibitor or low-dose rivaroxaban) to aspirin for long-term secondary prevention should be considered in patients with a high risk of ischaemic events and without high bleeding risk	Figure 7 incorporates patient preferences into the algorithm Includes link to: www.u-prevent.com	3	D
ESC 2021 Cardiovascular disease prevention	Adding a second antithrombotic drug to aspirin for long-term secondary prevention may be considered in patients with a moderate risk of ischaemic events and without a high bleeding risk	Figure 7 incorporates patient preferences into the algorithm Includes link to: www.u-prevent.com	3	D

© 2023 MacDonald BJ et al. *JAMA Network Open.*

Guideline (Year of Publication)	Recommendation	Supporting Text Incorporating Shared Decision Making	Directness	Facilitation
ESC 2021 Cardiovascular disease prevention	In patients with CCS, clopidogrel 75 mg daily is recommended, in addition to aspirin, for 6 months following coronary stenting, irrespective of stent type, unless a shorter duration (1 - 3 months) is indicated due to risk or occurrence of life-threatening bleeding.	Figure 7 incorporates patient preferences into the algorithm Includes link to: www.u-prevent.com	3	D
ESC 2021 Cardiovascular disease prevention	In ACS, DAPT with a P2Y12 inhibitor in addition to aspirin is recommended for 12 months, unless there are contraindications such as excessive risk of bleeding	Figure 7 incorporates patient preferences into the algorithm Includes link to: www.u-prevent.com	3	D
ESC 2022 Cardio-oncology	Long-term anticoagulation is recommended for stroke/systemic thromboembolism prevention in patients with cancer with AF and a CHA2DS2-VASc score ≥2 (men) or ≥3 (women) as per the 2020 ESC Guidelines for the diagnosis and management of atrial fibrillation	In the absence of direct evidence, anticoagulation to prevent thromboembolic events should be considered in patients at risk for stroke with AF after cancer surgery considering the anticipated net clinical benefit and informed patient preferences.	3	D
ESC 2022 Cardio-oncology	Long-term anticoagulation should be considered for stroke/systemic thromboembolism prevention in patients with cancer with AF and a CHA2DS2-VASc score=1 (men) or=2 (women) as per the 2020 ESC Guidelines for the diagnosis and management of atrial fibrillation	In the absence of direct evidence, anticoagulation to prevent thromboembolic events should be considered in patients at risk for stroke with AF after cancer surgery considering the anticipated net clinical benefit and informed patient preferences.	3	D
ESC 2022 Cardio-oncology	Patients with cancer, AF, and CHA2DS2-VASc score 0 (men) or 1 (women) may have a higher thrombotic risk than patients without cancer and may be considered for therapeutic anticoagulation after consideration of the bleeding risk	In the absence of direct evidence, anticoagulation to prevent thromboembolic events should be considered in patients at risk for stroke with AF after cancer surgery considering the anticipated net clinical benefit and informed patient preferences.	3	D
ESC 2022 Cardio-oncology	NOAC should be considered for stroke prevention in preference to LMWH and VKA (excluding patients with mechanical heart valves or moderate-to-severe mitral stenosis) in patients without a high bleeding risk, significant drug–drug interactions, or severe renal dysfunction	In the absence of direct evidence, anticoagulation to prevent thromboembolic events should be considered in patients at risk for stroke with AF after cancer surgery considering the anticipated net clinical benefit and informed patient preferences.	3	D
ESC 2022 Cardio-oncology	LMWH should be considered in patients with active cancer and AF who are not suitable for NOAC	In the absence of direct evidence, anticoagulation to prevent thromboembolic events should be considered in patients at risk for stroke with AF after cancer surgery considering the anticipated net clinical benefit and informed patient preferences.	3	D
ESC 2022 Cardio-oncology	Antiplatelet therapy or prophylactic LMWH are not recommended for stroke or systemic thromboembolism prevention in AF with cancer	In the absence of direct evidence, anticoagulation to prevent thromboembolic events should be considered in patients at risk	3	D

© 2023 MacDonald BJ et al. *JAMA Network Open.*

Guideline (Year of Publication)	Recommendation	Supporting Text Incorporating Shared Decision Making	Directness	Facilitation
		for stroke with AF after cancer surgery considering the anticipated net clinical benefit and informed patient preferences.		
ESC 2022 Cardio-oncology	A temporary interruption of cancer therapy is recommended in patients where the cancer therapy is suspected as a contributing cause.	Cancer treatment should be temporarily interrupted, and an urgent multidisciplinary approach is indicated to plan an individualized guideline-based management, taking into account cancer status, prognosis, and the patient's preferences regarding invasive management.	3	D
ESC 2022 Cardio-oncology	Apixaban, edoxaban, or rivaroxaban are recommended for the treatment of symptomatic or incidental VTE in patients with cancer without contraindications	A proposed approach to anticoagulation therapy in cancer-associated venous thrombosis, based on the TBIP acronym (Thromboembolic risk, Bleeding risk, drug–drug Interactions, Patient preferences)... Shared decision-making considering informed patient preferences should guide the choice of anticoagulation.	3	D
ESC 2022 Cardio-oncology	LMWH are recommended for the treatment of symptomatic or incidental VTE in patients with cancer with platelet count >50 000/μL	A proposed approach to anticoagulation therapy in cancer-associated venous thrombosis, based on the TBIP acronym (Thromboembolic risk, Bleeding risk, drug–drug Interactions, Patient preferences)... Shared decision-making considering informed patient preferences should guide the choice of anticoagulation.	3	D
ESC 2022 Cardio-oncology	In patients with cancer with platelet counts of 25 000–50 000/μL, anticoagulation with half-dose LMWH may be considered after a multidisciplinary discussion	A proposed approach to anticoagulation therapy in cancer-associated venous thrombosis, based on the TBIP acronym (Thromboembolic risk, Bleeding risk, drug–drug Interactions, Patient preferences)... Shared decision-making considering informed patient preferences should guide the choice of anticoagulation.	3	D
ESC 2022 Cardio-oncology	Prolongation of anticoagulation therapy beyond 6 months should be considered in selected patients with active cancer including metastatic disease	A proposed approach to anticoagulation therapy in cancer-associated venous thrombosis, based on the TBIP acronym (Thromboembolic risk, Bleeding risk, drug–drug Interactions, Patient preferences)... Shared decision-making considering informed patient preferences should guide the choice of anticoagulation.	3	D
ESC 2022 Cardio-oncology	Duration of anticoagulation in patients with cancer with a catheter-associated VTE is recommended for a minimum of 3 months and continuing longer if the catheter remains in situ.	A proposed approach to anticoagulation therapy in cancer-associated venous thrombosis, based on the TBIP acronym (Thromboembolic risk, Bleeding risk, drug–drug Interactions, Patient preferences)... Shared decision-making considering informed patient preferences should guide the choice of anticoagulation.	3	D
ACC 2012 Stable Ischemic Heart Disease	Initiation of pharmacotherapy interventions to achieve target HbA1c might be reasonable	A goal HbA1c 7%—a level approximating that achieved in the intensive-therapy arms of the DCCT, UKPDS, and PROactive studies—is reasonable for many younger patients, depending on	3	D

© 2023 MacDonald BJ et al. *JAMA Network Open.*

Guideline (Year of Publication)	Recommendation	Supporting Text Incorporating Shared Decision Making	Directness	Facilitation
		their duration of diabetes mellitus, comorbidities, adherence, and personal preferences		
ACC 2012 STEMI	The duration of triple antithrombotic therapy with a vitamin K antagonist, aspirin, and a P2Y12 receptor inhibitor should be minimized to the extent possible to limit the risk of bleeding	Triple therapy with a vitamin K antagonist, aspirin, and a P2Y12 receptor inhibitor should be restricted to specific clinical situations after STEMI in which the risk of systemic or venous thromboembolism or stent thrombosis is considered to exceed that of bleeding. Patient preferences and values should be taken into consideration, because individuals may weigh these outcomes differently	3	D
ACC 2014 NSTEMI	In addition to aspirin, a P2Y12 inhibitor (either clopidogrel or ticagrelor) should be continued for up to 12 months in all patients with NSTE-ACS without contraindications who are treated with an ischemia-guided strategy. Options include: Clopidogrel: 75mg daily	In patients with NSTE-ACS in whom there are indications for triple therapy, the benefit of such therapy in terms of prevention of stent thrombosis, thromboembolic events, and recurrent MI must be weighed against the risk of bleeding complications. Similarly, DAPT, in addition to anticoagulant therapy, requires consideration of the increased risk of bleeding. It is essential that therapeutic decision making in this critical area include discussion with the patient about the options, advantages, and limitations of available approaches.	3	D
ACC 2014 NSTEMI	In addition to aspirin, a P2Y12 inhibitor (either clopidogrel or ticagrelor) should be continued for up to 12 months in all patients with NSTE-ACS without contraindications who are treated with an ischemia-guided strategy. Options include: Ticagrelor: 90 mg twice daily	In patients with NSTE-ACS in whom there are indications for triple therapy, the benefit of such therapy in terms of prevention of stent thrombosis, thromboembolic events, and recurrent MI must be weighed against the risk of bleeding complications. Similarly, DAPT, in addition to anticoagulant therapy, requires consideration of the increased risk of bleeding. It is essential that therapeutic decision making in this critical area include discussion with the patient about the options, advantages, and limitations of available approaches.	3	D
ACC 2014 NSTEMI	In patients receiving a stent (bare-metal stent or DES) during PCI for NSTE-ACS, P2Y12 inhibitor therapy should be given for at least 12 months. Options include: Clopidogrel: 75 mg daily	In patients with NSTE-ACS in whom there are indications for triple therapy, the benefit of such therapy in terms of prevention of stent thrombosis, thromboembolic events, and recurrent MI must be weighed against the risk of bleeding complications. Similarly, DAPT, in addition to anticoagulant therapy, requires consideration of the increased risk of bleeding. It is essential that therapeutic decision making in this critical area include discussion with the patient about the options, advantages, and limitations of available approaches.	3	D
ACC 2014 NSTEMI	In patients receiving a stent (bare-metal stent or DES) during PCI for NSTE-ACS, P2Y12 inhibitor therapy should be given for at least 12 months. Options include: Prasugrel: 10 mg daily	In patients with NSTE-ACS in whom there are indications for triple therapy, the benefit of such therapy in terms of prevention of stent thrombosis, thromboembolic events, and recurrent MI must be weighed against the risk of bleeding complications. Similarly, DAPT, in addition to anticoagulant therapy, requires consideration of the increased risk of bleeding. It is essential that	3	D

© 2023 MacDonald BJ et al. *JAMA Network Open.*

Guideline (Year of Publication)	Recommendation	Supporting Text Incorporating Shared Decision Making	Directness	Facilitation
		therapeutic decision making in this critical area include discussion with the patient about the options, advantages, and limitations of available approaches.		
ACC 2014 NSTEMI	In patients receiving a stent (bare-metal stent or DES) during PCI for NSTE-ACS, P2Y12 inhibitor therapy should be given for at least 12 months. Options include: Ticagrelor: 90mg twice daily	In patients with NSTE-ACS in whom there are indications for triple therapy, the benefit of such therapy in terms of prevention of stent thrombosis, thromboembolic events, and recurrent MI must be weighed against the risk of bleeding complications. Similarly, DAPT, in addition to anticoagulant therapy, requires consideration of the increased risk of bleeding. It is essential that therapeutic decision making in this critical area include discussion with the patient about the options, advantages, and limitations of available approaches.	3	D
ACC 2014 NSTEMI	It is reasonable to use ticagrelor in preference to clopidogrel for maintenance P2Y12 treatment in patients with NSTE-ACS who undergo an early invasive or ischemia-guided strategy	In patients with NSTE-ACS in whom there are indications for triple therapy, the benefit of such therapy in terms of prevention of stent thrombosis, thromboembolic events, and recurrent MI must be weighed against the risk of bleeding complications. Similarly, DAPT, in addition to anticoagulant therapy, requires consideration of the increased risk of bleeding. It is essential that therapeutic decision making in this critical area include discussion with the patient about the options, advantages, and limitations of available approaches.	3	D
ACC 2014 NSTEMI	If the risk of morbidity from bleeding outweighs the anticipated benefit of a recommended duration of P2Y12 inhibitor therapy after stent implantation, earlier discontinuation (e.g., <12 months) of P2Y12 inhibitor therapy is reasonable	In patients with NSTE-ACS in whom there are indications for triple therapy, the benefit of such therapy in terms of prevention of stent thrombosis, thromboembolic events, and recurrent MI must be weighed against the risk of bleeding complications. Similarly, DAPT, in addition to anticoagulant therapy, requires consideration of the increased risk of bleeding. It is essential that therapeutic decision making in this critical area include discussion with the patient about the options, advantages, and limitations of available approaches.	3	D
ACC 2014 NSTEMI	Continuation of DAPT beyond 12 months may be considered in patients undergoing stent implantation	In patients with NSTE-ACS in whom there are indications for triple therapy, the benefit of such therapy in terms of prevention of stent thrombosis, thromboembolic events, and recurrent MI must be weighed against the risk of bleeding complications. Similarly, DAPT, in addition to anticoagulant therapy, requires consideration of the increased risk of bleeding. It is essential that therapeutic decision making in this critical area include discussion with the patient about the options, advantages, and limitations of available approaches.	3	D
ACC 2014 NSTEMI	The duration of triple antithrombotic therapy with a vitamin K antagonist, aspirin, and a P2Y12 receptor inhibitor in patients with NSTE-ACS	In patients with NSTE-ACS in whom there are indications for triple therapy, the benefit of such therapy in terms of prevention of stent thrombosis, thromboembolic events, and recurrent MI	3	D

© 2023 MacDonald BJ et al. *JAMA Network Open.*

Guideline (Year of Publication)	Recommendation	Supporting Text Incorporating Shared Decision Making	Directness	Facilitation
	should be minimized to the extent possible to limit the risk of bleeding.	must be weighed against the risk of bleeding complications. Similarly, DAPT, in addition to anticoagulant therapy, requires consideration of the increased risk of bleeding. It is essential that therapeutic decision making in this critical area include discussion with the patient about the options, advantages, and limitations of available approaches.		
ACC 2014 NSTEMI	Targeting oral anticoagulant therapy to a lower international normalized ratio (INR) (e.g., 2.0 to 2.5) may be reasonable in patients with NSTE-ACS managed with aspirin and a P2Y12 inhibitor.	In patients with NSTE-ACS in whom there are indications for triple therapy, the benefit of such therapy in terms of prevention of stent thrombosis, thromboembolic events, and recurrent MI must be weighed against the risk of bleeding complications. Similarly, DAPT, in addition to anticoagulant therapy, requires consideration of the increased risk of bleeding. It is essential that therapeutic decision making in this critical area include discussion with the patient about the options, advantages, and limitations of available approaches.	3	D
ACC 2014 NSTEMI	Older patients with NSTE-ACS should be treated with GDMT, an early invasive strategy, and revascularization as appropriate	Management decisions for older patients with NSTE-ACS should be patient centered, and consider patient preferences/ goals, comorbidities, functional and cognitive status, and life expectancy	3	D
ACC 2014 NSTEMI	Pharmacotherapy in older patients with NSTE-ACS should be individualized and dose adjusted by weight and/or CrCl to reduce adverse events caused by age-related changes in pharmacokinetics/dynamics, volume of distribution, comorbidities, drug interactions, and increased drug sensitivity	Management decisions for older patients with NSTE-ACS should be patient centered, and consider patient preferences/ goals, comorbidities, functional and cognitive status, and life expectancy	3	D
ACC 2014 NSTEMI	Bivalirudin, rather than a GP IIb/IIIa inhibitor plus UFH, is reasonable in older patients with NSTE-ACS, both initially and at PCI, given similar efficacy but less bleeding risk	Management decisions for older patients with NSTE-ACS should be patient centered, and consider patient preferences/ goals, comorbidities, functional and cognitive status, and life expectancy	3	D
ACC 2014 Perioperative	Neuraxial anesthesia for postoperative pain relief can be effective in patients undergoing abdominal aortic surgery to decrease the incidence of perioperative MI	In patients for whom neuraxial anesthesia (epidural or spinal anesthesia) is an option as the primary anesthetic or as a supplement to general anesthesia, several factors, such as the type of surgery, patient comorbidities, and patient preferences, are crucial in determining risk versus benefits.	3	D
ACC 2016 Supraventricular Tachycardia	Oral beta blockers, diltiazem, or verapamil is useful for ongoing management in patients with symptomatic SVT who do not have ventricular pre-excitation during sinus rhythm	The recommendations and algorithm (Figure 9) for ongoing management, along with other recommendations and algorithms for specific SVTs that follow, are meant to include consideration of patient preferences and clinical judgment	3	D
ACC 2016 Supraventricular Tachycardia	Flecainide or propafenone is reasonable for ongoing management in patients without structural heart disease or ischemic heart	The recommendations and algorithm (Figure 9) for ongoing management, along with other recommendations and algorithms	3	D

© 2023 MacDonald BJ et al. *JAMA Network Open.*

Guideline (Year of Publication)	Recommendation	Supporting Text Incorporating Shared Decision Making	Directness	Facilitation
	disease who have symptomatic SVT and are not candidates for, or prefer not to undergo, catheter ablation	for specific SVTs that follow, are meant to include consideration of patient preferences and clinical judgment		
ACC 2016 Supraventricular Tachycardia	Sotalol may be reasonable for ongoing management in patients with symptomatic SVT who are not candidates for, or prefer not to undergo, catheter ablation	The recommendations and algorithm (Figure 9) for ongoing management, along with other recommendations and algorithms for specific SVTs that follow, are meant to include consideration of patient preferences and clinical judgment	3	D
ACC 2016 Supraventricular Tachycardia	Dofetilide may be reasonable for ongoing management in patients with symptomatic SVT who are not candidates for, or prefer not to undergo, catheter ablation and in whom beta blockers, diltiazem, flecainide, propafenone, or verapamil are ineffective or contraindicated	The recommendations and algorithm (Figure 9) for ongoing management, along with other recommendations and algorithms for specific SVTs that follow, are meant to include consideration of patient preferences and clinical judgment	3	D
ACC 2016 Supraventricular Tachycardia	Oral amiodarone may be considered for ongoing management in patients with symptomatic SVT who are not candidates for, or prefer not to undergo, catheter ablation and in whom beta blockers, diltiazem, dofetilide, flecainide, propafenone, sotalol, or verapamil are ineffective or contraindicated	The recommendations and algorithm (Figure 9) for ongoing management, along with other recommendations and algorithms for specific SVTs that follow, are meant to include consideration of patient preferences and clinical judgment	3	D
ACC 2016 Supraventricular Tachycardia	Oral digoxin may be reasonable for ongoing management in patients with symptomatic SVT without preexcitation who are not candidates for, or prefer not to undergo, catheter ablation	The recommendations and algorithm (Figure 9) for ongoing management, along with other recommendations and algorithms for specific SVTs that follow, are meant to include consideration of patient preferences and clinical judgment	3	D
ACC 2016 DAPT with CAD	In patients with SIHD treated with DAPT after BMS implantation, P2Y12 inhibitor therapy (clopidogrel) should be given for a minimum of 1 month	DAPT is not recommended in patients with SIHD without prior stent implantation and no history of ACS or MI. Decisions about treatment with and duration of DAPT in patients with SIHD with a history of MI or coronary stent implantation require a thoughtful assessment of the benefit/risk ratio, integration of study data, and consideration of patient preference	3	D
ACC 2016 DAPT with CAD	In patients with SIHD treated with DAPT after DES implantation, P2Y12 inhibitor therapy (clopidogrel) should be given for at least 6 months	DAPT is not recommended in patients with SIHD without prior stent implantation and no history of ACS or MI. Decisions about treatment with and duration of DAPT in patients with SIHD with a history of MI or coronary stent implantation require a thoughtful assessment of the benefit/risk ratio, integration of study data, and consideration of patient preference	3	D
ACC 2016 DAPT with CAD	In patients with SIHD treated with DAPT after BMS or DES implantation who have tolerated DAPT without a bleeding complication and who are not at high bleeding risk (e.g., prior bleeding on DAPT, coagulopathy, oral anticoagulant use), continuation of DAPT with clopidogrel for longer	DAPT is not recommended in patients with SIHD without prior stent implantation and no history of ACS or MI. Decisions about treatment with and duration of DAPT in patients with SIHD with a history of MI or coronary stent implantation require a thoughtful assessment of the benefit/risk ratio, integration of study data, and consideration of patient preference	3	D

© 2023 MacDonald BJ et al. *JAMA Network Open.*

Guideline (Year of Publication)	Recommendation	Supporting Text Incorporating Shared Decision Making	Directness	Facilitation
	than 1 month in patients treated with BMS or longer than 6 months in patients treated with DES may be reasonable			
ACC 2016 DAPT with CAD	In patients with SIHD treated with DAPT after DES implantation who develop a high risk of bleeding (e.g., treatment with oral anticoagulant therapy), are at high risk of severe bleeding complication (e.g., major intracranial surgery), or develop significant overt bleeding, discontinuation of P2Y12 inhibitor therapy after 3 months may be reasonable	DAPT is not recommended in patients with SIHD without prior stent implantation and no history of ACS or MI. Decisions about treatment with and duration of DAPT in patients with SIHD with a history of MI or coronary stent implantation require a thoughtful assessment of the benefit/risk ratio, integration of study data, and consideration of patient preference	3	D
ACC 2018 Hypertension	Adults with HFpEF and persistent hypertension after management of volume overload should be prescribed ACE inhibitors or ARBs and beta blockers titrated to attain SBP of less than 130 mm Hg	[HFpEF section specifically] A shared decision-making discussion, with the patient influenced by clinician judgment, should drive the ultimate choice of antihypertensive agents.	3	D
ACC 2018 Hypertension	For initiation of antihypertensive drug therapy, first-line agents include thiazide diuretics, CCBs, and ACE inhibitors or ARBs	Initial drug selections should be based on trial evidence of treatment efficacy, combined with recognition of compelling indications for use of an agent from a specific drug class, as well as the individual patient's lifestyle preferences and traits	3	D
ACC 2018 Hypertension	Initiation of antihypertensive drug therapy with 2 first-line agents of different classes, either as separate agents or in a fixed-dose combination, is recommended in adults with stage 2 hypertension and an average BP more than 20/10 mm Hg above their BP target	Consideration of patient comorbidities, lifestyle, and preferences may suggest better tolerance or greater effect from one class of medication versus other classes	3	D
ACC 2019 Cholesterol	In patients 30 to 75 years of age with heterozygous FH and with an LDL-C level of 100 mg/dL or higher (≥2.6 mmol/L) while taking maximally tolerated statin and ezetimibe therapy, the addition of a PCSK9 inhibitor may be considered	the use of PCSK9 inhibitors in selected maximally treated patients with heterozygous FH with persistently elevated LDL-C levels may be considered after a clinician–patient discussion of the net benefits versus the cost of such therapy.	3	D
ACC 2019 Cholesterol	In patients 40 to 75 years of age with a baseline LDL-C level of 220 mg/dL or higher (≥5.7 mmol/L) and who achieve an on-treatment LDL-C level of 130 mg/dL or higher (≥3.4 mmol/L) while receiving maximally tolerated statin and ezetimibe therapy, the addition of a PCSK9 inhibitor may be considered	Regardless of whether a patient with LDL-C levels ≥190 mg/dL (≥4.9 mmol/L) is found to have a genetic mutation associated with FH, those with very high LDL-C values are most likely to achieve the greatest benefit from evidence-based LDL-C–lowering therapy. Consequently, patients who have a baseline LDL-C level ≥220 mg/dL (≥5.7 mmol/L) and an on-treatment LDL-C level ≥130 mg/dL (≥3.4 mmol/L) despite maximally tolerated statin and ezetimibe therapy may be considered for treatment with a PCSK9 inhibitor after a clinician–patient discussion of the net benefits versus the costs of such therapy.	3	D

© 2023 MacDonald BJ et al. *JAMA Network Open.*

Guideline (Year of Publication)	Recommendation	Supporting Text Incorporating Shared Decision Making	Directness	Facilitation
ACC 2019 Cholesterol	In adults with diabetes mellitus and 10-year ASCVD risk of 20% or higher, it may be reasonable to add ezetimibe to maximally tolerated statin therapy to reduce LDL-C levels by 50% or more	Therefore, a risk discussion may be held on the benefits of achieving ≥50% LDL-C lowering in adults with diabetes mellitus who have ≥20% ASCVD risk.	3	D
ACC 2019 Cholesterol	In adults 20 to 39 years of age with diabetes mellitus that is either of long duration (≥10 years of type 2 diabetes mellitus, ≥20 years of type 1 diabetes mellitus), albuminuria (≥30 mcg of albumin/mg creatinine), estimated glomerular filtration rate (eGFR) less than 60 mL/min/1.73 m2, retinopathy, neuropathy, or ankle-brachial index (ABI; <0.9), it may be reasonable to initiate statin therapy	Thus, it may be reasonable to have a discussion about initiating moderate-intensity statin therapy with patients who have had type 2 diabetes mellitus for at least 10 years or type 1 diabetes mellitus for at least 20 years and with patients with 1 or more major CVD risk factors or complications, such as diabetic retinopathy (S4.3-19), neuropathy (S4.3-16), nephropathy (eGFR <60 mL/min/1.73 m2 or albuminuria $30 mcg albumin/mg creatinine) (S4.3-25), or an ABI of <0.9... For patients >75 years of age, RCT evidence for statin therapy is not strong, so clinical assessment of risk status in a clinician–patient risk discussion is needed for deciding whether to continue or initiate statin treatment	3	D
ACC 2019 Cholesterol	In intermediate-risk patients, LDL-C levels should be reduced by 30% or more, and for optimal ASCVD risk reduction, especially in high-risk patients, levels should be reduced by 50% or more	If in the context of a risk discussion, maximal ASCVD risk reduction is desired, it is reasonable to use a high-intensity statin to lower LDL-C by ≥50%	3	D
ACC 2019 Cholesterol	For the primary prevention of clinical ASCVD* in adults 40 to 75 years of age without diabetes mellitus and with an LDL-C level of 70 to 189 mg/dL (1.7 to 4.8 mmol/L), the 10-year ASCVD risk of a first "hard" ASCVD event (fatal and nonfatal MI or stroke) should be estimated by using the race- and sex-specific PCE, and adults should be categorized as being at low risk (<5%), borderline risk (5% to <7.5%), intermediate-risk (≥7.5% to <20%), and high-risk (≥20%)	In individuals 40 to 75 years of age, 10-year ASCVD risk assessment begins the clinician–patient risk discussion... he potential for errors in estimating ASCVD risk at both ends of the risk curve (low risk and high-risk) as noted for individuals can be reviewed in the clinician–patient risk discussion	3	D
ACC 2019 Cholesterol	In intermediate-risk adults or selected borderline-risk adults in whom a CAC score is measured for the purpose of making a treatment decision, AND<br>- If the coronary calcium score is zero, it is reasonable to withhold statin therapy and reassess in 5 to 10 years, as long as higher risk conditions are absent (diabetes mellitus, family history of premature CHD, cigarette smoking);	Therefore, for patients with CAC scores of 1 to 99, it is reasonable to repeat the risk discussion.	3	D

© 2023 MacDonald BJ et al. *JAMA Network Open.*

Guideline (Year of Publication)	Recommendation	Supporting Text Incorporating Shared Decision Making	Directness	Facilitation
	- If CAC score is 1 to 99, it is reasonable to initiate statin therapy for patients ≥55 years of age;<br>- If CAC score is 100 or higher or in the 75th percentile or higher, it is reasonable to initiate statin therapy			
ACC 2019 Cholesterol	In intermediate-risk adults who would benefit from more aggressive LDL-C lowering and in whom high intensity- statins are advisable but not acceptable or tolerated, it may be reasonable to add a non-statin drug (ezetimibe or bile acid sequestrant) to a moderate-intensity statin	These therapies should be considered in the context of a risk discussion that reviews potential for benefit along with tolerability and safety issues.	3	D
ACC 2019 Cholesterol	In adults 75 years of age or older, it may be reasonable to stop statin therapy when functional decline (physical or cognitive), multimorbidity, frailty, or reduced life-expectancy limits the potential benefits of statin therapy	Nonetheless, these studies also show that decisions about statins are not intuitive because many frailer or more complex patients may prefer to stay on statins precisely because they are at greatest cardiovascular risk. Therefore, it is warranted that decisions about statin therapy be individualized and derived from clinician–patient discussions. Moreover, given the predictable fluctuations of health dynamics, such shared decisions should be reconsidered regularly.	3	D
ACC 2019 Cholesterol	Clinicians should consider conditions specific to women, such as premature menopause (age <40 years) and history of pregnancy-associated disorders (hypertension, preeclampsia, gestational diabetes mellitus, small-for-gestational-age infants, preterm deliveries), when discussing lifestyle intervention and the potential for benefit of statin therapy	Decisions should be made in the context of a risk discussion and should take into consideration an informed patient preferences	3	D
ACC 2019 Cholesterol	In adults 76 to 80 years of age with an LDL-C level of 70 to 189 mg/dL (1.7 to 4.8 mmol/L), it may be reasonable to measure CAC to reclassify those with a CAC score of zero to avoid statin therapy	Limiting statin therapy to those with CAC scores greater than zero, combined with clinical judgment and patient preference, could provide a valuable awareness with which to inform shared decision-making.	3	D
ACC 2019 Cholesterol	In children and adolescents 10 years of age or older with an LDL-C level persistently 190 mg/dL or higher (≥4.9 mmol/L) or 160 mg/dL or higher (≥4.1 mmol/L) with a clinical presentation consistent with FH (see Section 4.2.) and who do not respond adequately with 3 to 6 months of lifestyle therapy, it is reasonable to initiate statin therapy	The intensity of treatment should be based on the severity of the hypercholesterolemia and should incorporate patient/family preference	3	D

© 2023 MacDonald BJ et al. *JAMA Network Open.*

Guideline (Year of Publication)	Recommendation	Supporting Text Incorporating Shared Decision Making	Directness	Facilitation
ACC 2019 Primary prevention	In intermediate risk (≥7.5% to <20% 10-year ASCVD risk) patients, LDL-C levels should be reduced by 30% or more, and for optimal ASCVD risk reduction, especially in patients at high risk (≥20% 10-year ASCVD risk), levels should be reduced by 50% or more	If in the context of a risk discussion, maximal ASCVD risk reduction is desired, it is reasonable to use a high-intensity statin to lower LDL-C by ≥50%.	3	D
ACC 2019 Primary prevention	In intermediate-risk (≥7.5% to <20% 10-year ASCVD risk) adults or selected borderline-risk (5% to <7.5% 10-year ASCVD risk) adults in whom a coronary artery calcium score is measured for the purpose of making a treatment decision, AND<br>- If the coronary artery calcium score is zero, it is reasonable to withhold statin therapy and reassess in 5 to 10 years, as long as higher-risk conditions are absent (e.g., diabetes, family history of premature CHD, cigarette smoking);<br>- If coronary artery calcium score is 1 to 99, it is reasonable to initiate statin therapy for patients ≥55 years of age;<br>- If coronary artery calcium	Therefore, for patients with coronary artery calcium scores of 1 to 99, it is reasonable to repeat the risk discussion	3	D
ACC 2019 Primary prevention	In adults who use tobacco, a combination of behavioral interventions plus pharmacotherapy is recommended to maximize quit rates	The best and most effective treatments are those that are acceptable to and feasible for an individual patient; clinicians should consider the patient's specific medical history and preferences and offer to provide tailored strategies that work best for the patient	3	D
ACC 2019 Primary prevention	Low-dose aspirin (75-100 mg orally daily) might be considered for the primary prevention of ASCVD among select adults 40 to 70 years of age who are at higher ASCVD risk but not at increased bleeding risk	These changes reflect the need to instead consider the totality of available evidence for ASCVD risk [inclusive, where appropriate, of risk-enhancing factors, such as strong family history of premature MI, inability to achieve lipid or BP or glucose targets, or significant elevation in coronary artery calcium score (S4.6-21)] and to also tailor decisions about prophylactic aspirin to patient and clinician preferences.	3	D
ACC 2022 Heart failure	In women with HF or cardiomyopathy who are pregnant or currently planning for pregnancy, ACEi, ARB, ARNi, MRA, SGLT2i, ivabradine, and vericiguat should not be administered because of significant risks of fetal harm	HFrEF medications considered acceptable during pregnancy (15), within a construct of multidisciplinary shared decision-making regarding benefits and potential risks, are furosemide, beta blockers (most commonly metoprolol) (63-65), hydralazine, and nitrates (13,14,19).	3	D
CCS 2018 Antiplatelet	In patients undergoing PCI who are treated with either a BMS or DES and who require elective noncardiac surgery, we suggest continuing ASA perioperatively whenever possible	Antiplatelet therapy management in the perioperative period should be based on a balanced assessment of the risks of coronary thrombotic complications vs the risk of perioperative	3	D

© 2023 MacDonald BJ et al. *JAMA Network Open.*

Guideline (Year of Publication)	Recommendation	Supporting Text Incorporating Shared Decision Making	Directness	Facilitation
		bleeding in discussion with the surgeon, interventional cardiologist, attending physician/cardiologist, and the patient.		
CCS 2018 Antiplatelet	In patients undergoing PCI who are treated with aBMS or DES and who have undergone noncardiac surgery, we suggest restarting maintenance-dose DAPT after surgery, as soon as it is deemed safe by the surgeon	Antiplatelet therapy management in the perioperative period should be based on a balanced assessment of the risks of coronary thrombotic complications vs the risk of perioperative bleeding in discussion with the surgeon, interventional cardiologist, attending physician/cardiologist, and the patient.	3	D
CCS 2018 Antiplatelet	We recommend continuation of ASA in all patients with ACS who require CABG surgery	Antiplatelet therapy management in the perioperative period should be based on a balanced assessment of the risks of coronary thrombotic complications vs the risk of perioperative bleeding in discussion with the surgeon, interventional cardiologist, attending physician/cardiologist, and the patient.	3	D
CCS 2018 Antiplatelet	To minimize the risk of bleeding, for patients with an ACS who are receiving ticagrelor and need semi-urgent CABG, we suggest a minimum interruption of ticagrelor for 48-72 hours before CABG	Antiplatelet therapy management in the perioperative period should be based on a balanced assessment of the risks of coronary thrombotic complications vs the risk of perioperative bleeding in discussion with the surgeon, interventional cardiologist, attending physician/cardiologist, and the patient.	3	D
CCS 2018 Antiplatelet	and recommend an ideal interruption period of 5 days before elective CABG	Antiplatelet therapy management in the perioperative period should be based on a balanced assessment of the risks of coronary thrombotic complications vs the risk of perioperative bleeding in discussion with the surgeon, interventional cardiologist, attending physician/cardiologist, and the patient.	3	D
CCS 2018 Antiplatelet	To minimize the risk of bleeding, for patients with an ACS who are receiving clopidogrel and need semi-urgent CABG, we suggest a minimum interruption of clopidogrel for 48-72 hours before CABG	Antiplatelet therapy management in the perioperative period should be based on a balanced assessment of the risks of coronary thrombotic complications vs the risk of perioperative bleeding in discussion with the surgeon, interventional cardiologist, attending physician/cardiologist, and the patient.	3	D
CCS 2018 Antiplatelet	and recommend an ideal interruption period of 5 days before elective CABG	Antiplatelet therapy management in the perioperative period should be based on a balanced assessment of the risks of coronary thrombotic complications vs the risk of perioperative bleeding in discussion with the surgeon, interventional cardiologist, attending physician/cardiologist, and the patient.	3	D
CCS 2018 Antiplatelet	To minimize the risk of bleeding, for patients with an ACS who are receiving prasugrel and need semi-urgent CABG, we suggest a minimum interruption of prasugrel for 5 days before CABG	Antiplatelet therapy management in the perioperative period should be based on a balanced assessment of the risks of coronary thrombotic complications vs the risk of perioperative bleeding in discussion with the surgeon, interventional cardiologist, attending physician/cardiologist, and the patient.	3	D
CCS 2018 Antiplatelet	and recommend an ideal interruption period of 7 days before elective CABG	Antiplatelet therapy management in the perioperative period should be based on a balanced assessment of the risks of coronary thrombotic complications vs the risk of perioperative bleeding in discussion with the surgeon, interventional cardiologist, attending physician/cardiologist, and the patient.	3	D

© 2023 MacDonald BJ et al. *JAMA Network Open.*

Guideline (Year of Publication)	Recommendation	Supporting Text Incorporating Shared Decision Making	Directness	Facilitation
CCS 2020 Atrial fibrillation	Stage 5 CKD (eGFR < 15mL/min or dialysis-dependent): we suggest that such patients not routinely receive antithrombotic therapy for stroke prevention in AF	Because of the lack of prospective data showing benefit in patients with a CrCl < 15 mL/min, the decision to use antithrombotic therapy should be individualized on the basis of physician and patient preference and considering the relative risks of stroke and bleeding.	3	D
CCS 2020 Atrial fibrillation	We suggest that patients with secondary AF, which has resolved, not be routinely anticoagulated in the absence of recurrence	This recommendation places high value on the recognition that secondary AF is often a self-limited process. In the absence of AF recurrence, the current evidence base is insufficient to recommend long-term OAC. However, some patients and providers might elect to pursue long-term OAC on the basis of assessment of the patients underlying risk of stroke and values and preferences.	3	D
CCS 2020 Atrial fibrillation	We recommend intermittent antiarrhythmic drug therapy ("pill-in-the-pocket") as an alternative to daily antiarrhythmic therapy in patients with infrequent, symptomatic episodes of AF	This recommendation is on the basis of the results of observational cohort studies that have shown efficacy and safety of intermittent antiarrhythmic drug therapy in selected patients. It places a high value on patient preferences and capabilities.	3	D
CCS 2021 Heart failure	We recommend preferentially use of drugs at target doses that have been proven to be beneficial in clinical trials as optimal medical therapy. If these doses cannot be achieved, the maximally tolerated dose is acceptable	The approach to initiation and titration of standard therapies should be directed by clinical and other patient factors including hemodynamic status, renal function, access to medication, adherence, anticipated side effects and tolerability, and patient preference.	3	D
CCS 2021 Dyslipidemia	We recommend intensification of lipid-lowering therapy with a PCSK9 inhibitor (evolocumab or alirocumab)— with or without the additional use of ezetimibe—for secondary CV prevention patients shown to derive the largest benefit from PCSK9 inhibitor therapy in whom LDL-C remains ≥ 1.8 mmol/L (or non-HDL-C ≥ 2.4 mmol/L or ApoB ≥ 0.7 g/L) while receiving the maximally tolerated statin dose	Clinicians should discuss the indication and potential benefits of a PCSK9 inhibitor with the patient, along with the coverage issues and the potential costs to them. Shared decision making remains key.	3	D
CCS 2021 Dyslipidemia	We recommend intensification of lipid-lowering therapy with ezetimibe and/or PCSK9 inhibitor therapy for all secondary prevention CVD patients in whom LDL-C remains ≥ 1.8 mmol/L (or non-HDL-C ≥ 2.4 mmol/L or ApoB ≥ 0.7 g/L) while receiving the maximally tolerated statin dose	Clinicians should discuss the indication and potential benefits of a PCSK9 inhibitor with the patient, along with the coverage issues and the potential costs to them. Shared decision making remains key.	3	D
CCS 2021 Dyslipidemia	We recommend the use of IPE to decrease the risk of CV events in patients with ASCVD, or with diabetes and ≥ 1 CVD risk factors, who have an elevated fasting triglyceride level of 1.5-5.6	As part of shared decision-making, clinicians should discuss the indication and potential benefits of IPE, as well as the coverage issues and the potential patient costs.	3	D

© 2023 MacDonald BJ et al. *JAMA Network Open.*

Guideline (Year of Publication)	Recommendation	Supporting Text Incorporating Shared Decision Making	Directness	Facilitation
	mmol/L despite treatment with maximally tolerated statin therapy			
CCS 2021 Dyslipidemia	We recommend the use of a PCSK9 inhibitor (alirocumab or evolocumab) to lower LDL-C level in patients with heterozygous FH without clinical ASCVD whose LDL-C remains above the target (ie, LDL-C ≥ 2.5 mmol/L or < 50% reduction from baseline; or ApoB ≥ 0.85 mg/dL or non-HDL-C ≥ 3.2 mmol/L) despite maximally tolerated statin therapy with or without ezetimibe therapy	The choice of agent should be on the basis of individual patient factors, their values and preferences, and practical considerations, such as access, cost, and adherence.	3	D
CCS 2021 Dyslipidemia	We recommend the use of a PCSK9 inhibitor (alirocumab or evolocumab) for patients with heterozygous FH and ASCVD whose LDL-C remains above the threshold ≥ 1.8 mmol/L (or ApoB ≥ 0.7 mg/dL or non-HDL-C ≥ 2.4 mmol/L) despite maximally tolerated statin therapy, with or without ezetimibe	The choice of agent should be on the basis of individual patient factors, their values and preferences, and practical considerations, such as access, cost, and adherence.	3	D
CCS 2022 PAD	We recommend that patients with PAD qualify as statin-indicated patients and should receive lipid-modifying therapy for the reduction of death, CV death, nonfatal MI, nonfatal stroke (MACE), and MALE concordant with the recommendations in the 2021 Canadian Cardiovascular Society (CCS) guidelines for the management of dyslipidemia<br>a. Maximally tolerated dose of statin therapy<br>b. Statin add-on therapies (ezetimibe and/or PCSK-9 inhibitors) if receiving maximally tolerated dose of statin therapy and the low-density lipoprotein cholesterol is ≥1.8 mmol/L, non-high-density lipoprotein cholesterol ≥2.4 mmol/L or apolipoprotein B100 ≥0.7 mg/dL.	Statin add-on therapy and icosapent ethyl present numerous challenges with respect to cost and access for many patients. They also contribute to the burden of medications and complexity of therapy. Any decision to implement them should be made through open patient-physician discussion.	3	D
CCS 2022 PAD	We recommend that patients with PAD, who, despite maximally tolerated dose of statin therapy have a triglyceride level of 1.5-5.6 mmol/L, should be considered for use of icosapent ethyl for the reduction CV death, nonfatal MI, and nonfatal stroke concordant with the recommendations in the 2021 CCS guidelines for the management of dyslipidemia	Statin add-on therapy and icosapent ethyl present numerous challenges with respect to cost and access for many patients. They also contribute to the burden of medications and complexity of therapy. Any decision to implement them should be made through open patient-physician discussion.	3	D
CCS 2022 PAD	We recommend that patients with PAD and type 2 diabetes should be offered a SGLT-2 inhibitor	The choice of antihyperglycemic agents in patients with PAD should be individualized to the patient's wishes, preferences,	3	D

© 2023 MacDonald BJ et al. *JAMA Network Open.*

Guideline (Year of Publication)	Recommendation	Supporting Text Incorporating Shared Decision Making	Directness	Facilitation
	compared with usual diabetic control because of the reduction in MACE without any risk of increased amputation	and financial support/drug coverage. However, diabetes medication should be chosen to provide the optimal CV protection and reduction in MALE		
CCS 2022 PAD	We suggest that patients with PAD and diabetes might benefit from use of a GLP-1 agonist or DPP-4 inhibitor	The choice of antihyperglycemic agents in patients with PAD should be individualized to the patient's wishes, preferences, and financial support/drug coverage. However, diabetes medication should be chosen to provide the optimal CV protection and reduction in MALE	3	D
CCS 2022 PAD	We recommend combination treatment with rivaroxaban 2.5 mg twice daily and aspirin or single antiplatelet therapy for patients with symptomatic lower extremity PAD and low bleeding risk in the absence of high-risk limb presentation or high-risk comorbidities	Patients who place a high value on minimizing ischemic risk, such as MI, stroke, acute limb ischemia, or major vascular amputation, might opt for rivaroxaban 2.5 mg twice daily in combination with aspirin. Patients who place a high value on bleeding avoidance and minimizing pill burden might opt for single antiplatelet therapy alone.	3	D
CCS 2022 PAD	We suggest that dual antiplatelet therapy (DAPT; aspirin and clopidogrel or aspirin and ticagrelor) be used for patients with symptomatic lower extremity PAD at high risk for vascular events, at low bleeding risk, and who have contraindications to rivaroxaban	The combination of ticagrelor and aspirin likely has greater ischemic benefit yet higher bleeding risk as contrasted with the combination of clopidogrel and aspirin, with choice of therapy directed by individual patient profile and preferences.	3	D

© 2023 MacDonald BJ et al. *JAMA Network Open.*

eTable 3. List of included guidelines and their characteristics

Category	Condition	Year	PDF page count	Main text word count	Number of authors (no COI declared)	General statement supporting SDM
**American College of Cardiology**						
Congenital, valvular, and aortic diseases	Adults with Congenital Heart Disease	2019	112	47,292	15 (7)	Yes
	Valvular Heart Disease	2021	173	73,625	15 (10)	Yes
	Aortic Disease	2022	171	70,549	29 (19)	Yes
Coronary artery disease	Stable Ischemic Heart Disease	2012	121	62,895	26 (10)	Yes
	STEMI	2013	63	29,172	23 (11)	Yes
	NSTEMI	2014	90	36,194	17 (11)	Yes
	DAPT with CAD (update)	2016	34	11,132	17 (10)	No
	Coronary artery revascularization	2022	109	39,190	24 (16)	Yes
Electrophysiology	Atrial fibrillation	2014	76	33,492	16 (10)	Yes
	Atrial fibrillation (update)	2019	29	10,266	15 (8)	Yes
	Supraventricular Tachycardia	2016	89	36,737	17 (11)	Yes
	Ventricular Arrhythmias and the Prevention of SCD	2018	130	52,511	19 (11)	Yes
	Bradycardia and Cardiac Conduction Delay	2018	106	43,362	19 (16)	Yes
General cardiology	Perioperative risk	2014	61	25,116	17 (12)	Yes
	PAD	2017	56	18,924	21 (12)	Yes
	Syncope	2017	72	25,973	16 (10)	Yes
	Hypertension	2018	122	46,563	21 (19)	Yes
	Cholesterol	2019	66	27,991	24 (0)	Yes
	Primary prevention	2019	56	17,905	18 (0)	Yes
Heart failure and myocardial disease	Hypertrophic Cardiomyopathy	2020	82	32,386	19 (10)	Yes
	Heart failure	2022	159	59,613	26 (14)	Yes
**Canadian Cardiovascular Society**						
Coronary artery disease	Refractory Angina	2012	22	8902	19 (11)	No
	Antiplatelet (update)	2013	12	5805	12 (NR)	No
	Antiplatelet therapy (update)	2018	20	9870	30 (9)	No
	Stable Ischemic Heart Disease	2014	13	5346	20 (NR)	No
	STEMI (update)	2019	34	11,940	34 (11)	No
Electrophysiology	Cardiac Resynchronization Therapy	2013	15	6847	17 (NR)	No
	Atrial fibrillation	2020	102	82,507	29 (6)	Yes
General cardiology	Cardio-oncology	2016	11	5479	25 (NR)	No
	Perioperative risk	2017	16	8888	13 (NR)	Yes
	Dyslipidemia	2021	22	11,238	26 (7)	Yes
	PAD	2022	28	14,985	37 (18)	No
	GLP1/SGLT2i	2022	15	7795	25 (NR)	Yes

© 2023 MacDonald BJ et al. *JAMA Network Open.*

Category	Condition	Year	PDF page count	Main text word count	Number of authors (no COI declared)	General statement supporting SDM
Heart failure and myocardial disease	Pediatric heart failure	2013	18	8518	23 (NR)	No
	Heart failure	2017	92	49,665	35 (NR)	No
	Heart failure (update)	2020	11	6723	40 (9)	No
	HFrEF (update)	2021	16	9634	40 (7)	No
**European Society of Cardiology**						
Congenital, valvular, and aortic diseases	Aortic diseases	2014	62	37,683	22 (4)	No
	Infective endocarditis	2015	54	30,499	20 (7)	No
	Adult congenital heart disease	2021	84	48,711	17 (3)	No
	Valvular heart disease	2022	72	32,099	21 (0)	No
Coronary artery disease	DAPT in CAD (update)	2018	48	25,238	17 (5)	No
	STEMI	2018	66	29,195	19 (4)	Yes
	Myocardial revascularization	2019	96	40,121	22 (3)	No
	Chronic coronary syndromes	2020	71	35,962	25 (3)	No
	NSTE-ACS	2021	79	36,367	24 (2)	No
General cardiology	Arterial hypertension	2018	98	52,334	28 (3)	No
	Cardiovascular disease during pregnancy	2018	84	56,693	20 (6)	No
	PAD	2018	60	27,503	23 (3)	No
	Syncope	2018	69	31,801	16 (7)	No
	Acute pulmonary embolism	2019	61	32,472	23 (1)	No
	Diabetes, pre-diabetes, CVD	2020	70	32,519	24 (2)	Yes
	Dyslipidemia	2020	78	43,424	21 (1)	No
	Cardiovascular disease prevention	2021	111	56,329	30 (6)	Yes
	Sports cardiology and exercise	2021	80	39,055	24 (8)	Yes
	Cardio-oncology	2022	133	56,399	30 (7)	No
	Cardiovascular assessment and manage of patients undergoing non-cardiac surgery	2022	99	48,585	29 (4)	Yes
	Pulmonary hypertension	2022	114	56,950	30 (3)	Yes
Electrophysiology	Atrial fibrillation	2020	126	48,949	24 (3)	Yes
	Supraventricular Tachycardia	2020	66	29,954	21 (2)	No
	Cardiac pacing and CRT	2021	94	42,839	24 (3)	Yes
	Ventricular Arrhythmias and the Prevention of SCD	2022	130	49,165	25 (6)	Yes
	Hypertrophic Cardiomyopathy	2014	55	25,714	20 (2)	No

© 2023 MacDonald BJ et al. *JAMA Network Open.*

Category	Condition	Year	PDF page count	Main text word count	Number of authors (no COI declared)	General statement supporting SDM
Heart failure and myocardial disease	Pericardial diseases	2015	44	24,551	18 (3)	No
	Heart failure	2021	128	56,828	31 (3)	No

CAD: Coronary artery disease, COI: Conflict of interest, CVD: Cardiovascular disease, CRT: Cardiac resynchronization therapy, DAPT: Dual antiplatelet therapy, GLP-1: Glucagon-like peptide-1, NR: Not reported, NSTE-ACS: Non-ST-segment elevation acute coronary syndrome, NSTEMI: Non-ST-Elevation Myocardial Infarction, PAD: Peripheral artery disease, SCD: Sudden cardiac death, SDM: Shared decision-making, SGLT2i: Sodium-glucose cotransporter-2 inhibitor, STEMI: ST-segment elevation myocardial infarction.

© 2023 MacDonald BJ et al. *JAMA Network Open.*
