# Supplementary material for: Incorporation of Shared Decision-Making in International Cardiovascular Guidelines, 2012-2022
Source: JAMA Netw Open. 2023 Sep 7;6(9):e2332793. doi: 10.1001/jamanetworkopen.2023.32793 (PMC10485733; doi:10.1001/jamanetworkopen.2023.32793)
Supplement: Supplement 2. — Data Sharing Statement [file jamanetwopen-e2332793-s002.pdf]

Data Sharing Statement

MacDonald. Incorporation of Shared Decision-Making in International Cardiovascular Guidelines, 2012-2022. *JAMA Netw Open*. Published September 07, 2023. doi:10.1001/jamanetworkopen.2023.32793

Data
Data available: Yes
Data types: Data (not involving human participants)
How to access data: All data used in this study are provided in the Supplemental Materials
When available: With publication

Supporting Documents
Document types: None

Additional Information
Who can access the data: This material will be available to anyone with access to the Supplemental Material
Types of analyses: For any purpose
Mechanisms of data availability: Without investigator support
